# Graphene Quantum Dot Solid Sheets: Strong blue-light-emitting & photocurrent-producing band-gap-opened nanostructures

**DOI:** 10.1038/s41598-017-10534-4

**Published:** 2017-09-07

**Authors:** Ganapathi Bharathi, Devaraj Nataraj, Sellan Premkumar, Murugaiyan Sowmiya, Kittusamy Senthilkumar, T. Daniel Thangadurai, Oleg Yu Khyzhun, Mukul Gupta, Deodatta Phase, Nirmalendu Patra, Shambhu Nath Jha, Dibyendu Bhattacharyya

**Affiliations:** 1 Low Dimensional Materials Laboratory, Department of Physics, Bharathiar University, Coimbatore, TN India; 2 Centre for Advanced Studies in Physics for the development of Solar Energy Materials and Devices, Department of Physics, Bharathiar University, Coimbatore, TN India; 3 Molecular Quantum Mechanics laboratory, Department of Physics, Bharathiar University, Coimbatore, TN India; 4 Department of Nanoscience and Technology, Sri Ramakrishna Engineering College, Coimbatore, TN India; 5 Department of Structural Chemistry of Solids, Frantsevych Institute for Problems of Materials Science, National Academy of Sciences of Ukraine, Kyiv, UA-03142 Ukraine; 6 UGC-DAE Consortium for Scientific Research, Indore, India; 7 Atomic & Molecular Physics Division, Bhabha Atomic Research Centre, Mumbai, India

## Abstract

Graphene has been studied intensively in opto-electronics, and its transport properties are well established. However, efforts to induce intrinsic optical properties are still in progress. Herein, we report the production of micron-sized sheets by interconnecting graphene quantum dots (GQDs), which are termed ‘GQD solid sheets’, with intrinsic absorption and emission properties. Since a GQD solid sheet is an interconnected QD system, it possesses the optical properties of GQDs. Metal atoms that interconnect the GQDs in the bottom-up hydrothermal growth process, induce the semiconducting behaviour in the GQD solid sheets. X-ray absorption measurements and quantum chemical calculations provide clear evidence for the metal-mediated growth process. The as-grown graphene quantum dot solids undergo a Forster Resonance Energy Transfer (FRET) interaction with GQDs to exhibit an unconventional 36% photoluminescence (PL) quantum yield in the blue region at 440 nm. A high-magnitude photocurrent was also induced in graphene quantum dot solid sheets by the energy transfer process.

## Introduction

Graphene is an interesting two-dimensional honeycomb carbon structure with very good electrical and thermal conductivities, strong chemical stability and high mechanical strength^1–6^. It can be prepared in different forms, such as sheets^7^, nanoribbons^8^, and graphene quantum dots (GQDs)^9^, by various methods. The size and shape, in turn, determine the properties and applications of graphene. For example, GQDs and graphene nanoribbons have photoluminescence properties^9, 10^, whereas graphene sheets do not, due to their metallic character^4^. While GQDs are finding applications in biology as biomarkers^11, 12^, graphene sheets are being used in optoelectronic device fabrication^13^. As far as preparation is concerned, graphene sheets are obtained by exfoliating graphite using various methods, which include Bordie’s and Hummer’s methods^14–19^. The chemical vapour deposition method is also used to prepare pristine graphene sheets^13^. In contrast, GQDs are obtained by top-down and bottom-up methods^9, 20^. A pristine graphene sheet is a zero-band-gap material due to the convergence of the valence band maximum and conduction band minimum at the Dirac point^21^. Photo-excited electron-hole pairs will undergo a non-radiative relaxation process through the continuum band states towards the band edge states, and therefore, photoluminescence is not possible. If a graphene sheet has to be used as an active layer in device fabrication, similar to other semiconducting materials, its band gap must be opened. By producing isolated domains of sp^2^ islands on the surface of graphene sheets, one can discretize the energy levels (molecular-like energy levels)^20^ so that the photo-excited electron–hole pairs will undergo radiative recombination through those discrete energy levels, resulting in an intrinsic emission. This intrinsic emission is an indication that the band gap has been opened in the graphene sheet sample. To produce such isolated sp^2^ domains on the surface of a graphene sheet, surface modifications/treatments involving either oxidation or partial reduction methods have been used^22, 23^. For example, H. Yoon *et al*. formed oxidation-controlled graphene quantum dots from graphite oxide that was prepared by the modified Hummer’s method and studied their intrinsic PL behaviour^24^. N. Amirhasan *et al*. used a plasma treatment on micromechanically exfoliated single-layer graphene to produce semiconducting graphene sheets and found broad green emission at approximately 550 nm^25^. In the reported case of plasma treated graphene sheets, a single-layer configuration is necessary to avoid the highly possible interlayer PL quenching effect; this is because the increase in the number of layers results in abrupt quenching of PL, as reported elsewhere^26^.

Overall observations indicate that it is always difficult to obtain strong intrinsic emission yield from a graphene sheet sample. In fact, there are no reports on the strong intrinsic emission behavior from graphene sheet samples, to the best of our knowledge. However, there are several reports that show the weak emission from graphene sheet samples is due to surface-attached molecules and is always excitation-energy dependent in nature^27, 28^. The best way to obtain a high emission yield from a graphene system is by preparing the samples in size-reduced form as QDs. In such smaller-sized GQDs, the sp^2^ domains are of nanoscale dimensions, and therefore, one can expect quantum size-effect-induced intrinsic emission to occur naturally. Mostly blue emission is reported from GQDs ranging from 1.8 to 7 nm^9^. The problem with GQDs is that not only the size affects the emission; (i) the edge effects (free zigzag sites/carbene states)^20^ and (ii) n-π* transitions from C=O and C=N bonds also contribute to the emission at 450 nm^9, 29^. For example, carbene-like states and the n-π* transition due to the absorption at 350 nm always show Stokes-shifted emissions at 450 nm, which matches the size-effect-induced emissions at 450 nm. Hence, it is always difficult to obtain a true size-effect-induced strong intrinsic emission from a GQD sample. There are few reports that claim to have obtained size-effect-induced emissions from GQDs^30^. Others have mostly attributed the reason for the emissions to n-π* transitions of C=O/C=N. Hence, there is an open challenge in graphene research to obtain high-yield photoluminescence that is purely of an intrinsic nature arising from the structure and not involving any functional groups. In the present work, we have successfully prepared GQD interconnected solid sheets that can be induced to give 36% intrinsic emission yield and a high photocurrent. This was possible because the surface effect of the GQDs was drastically reduced in the GQD interconnected solid sheets.

Recently, research on QD solids has attracted interest because one cannot use the as-prepared colloidal QDs for applications requiring electrical transport properties due to the poor inter-dot charge transport mechanism. By interconnecting the QDs together, one can prepare a solid system with the desired absorption and emission properties. It is even possible to prepare an alternative material to silicon by this method. However, the preparation of such QD solid structures is a challenging process and, to the best of our knowledge, only a few reports are available on the successful preparation of QD solid systems, mostly involving inorganic systems such as PbS, CdSe and PbSe^31–34^. As far as such QD solid preparation is concerned, different shaped/faceted nanocrystals made up of heavier elements were interconnected with lighter organic/inorganic groups by slow evaporation and ligand exchange processes^35^. Such inorganic QD solid systems are weakly coupled and sensitive to external parameters such as temperature, thus limiting the effective utilization of the QD solid systems. The strong coupling between QDs is required for the effective usage of QD solid systems in a wide range of applications such as terahertz lasing and quantum computing^35, 36^. In the present work, we have successfully prepared GQD solid sheet structures of a single-crystalline nature by interconnecting GQDs with metal atoms. Since the basic building block (GQD) is made up of a light element and is of planar dimensions, it was possible to interconnect them through heavier metal atoms, and thus a stronger coupling is established between the GQDs. Oxygen has also played an important role in the interconnection process. The isolated sp^2^ domains were created in the interconnection process, which in turn helped to retain the quantum behaviour of the individual dots in the solid sheet. As a consequence, a new material system of graphene with a band gap was made possible. In this present paper, we discuss these results in detail.

## Results

### Growth and optical properties of GQD solid sheets

**Figure 1:**
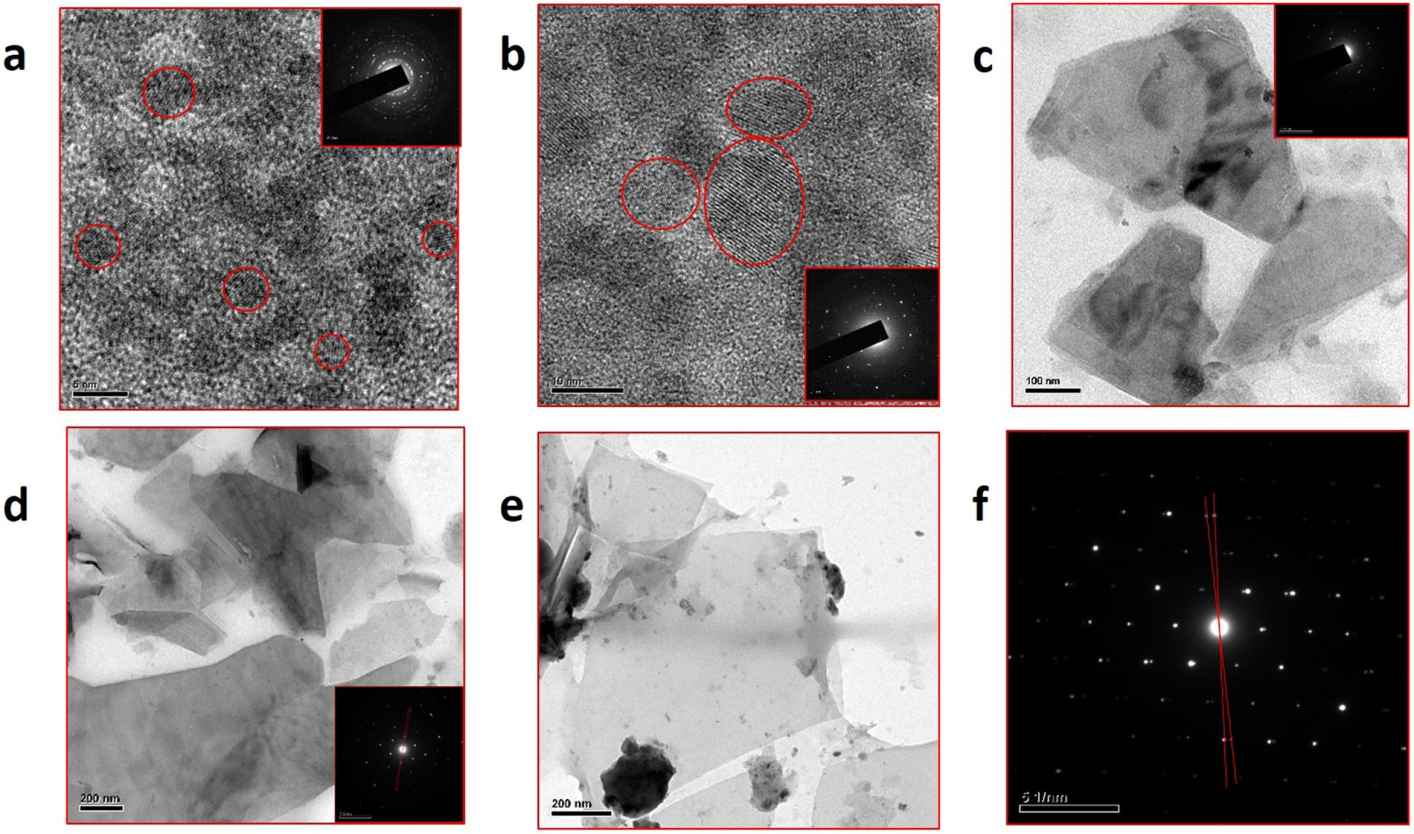
HRTEM analysis of GQDs and GQD solid sheet structures. (**a**) Spherical shaped GQDs with size around 5 nm from Z0 sample (**b**) spherical shaped GQDs with size around 10 nm from the Z1 sample (**c**) slab like GQD solid sheets in 100 nm size range from Z3 sample (**d–e**) slab like GQD solid sheets having average size about 1µm from Z5 and Z7 samples, respectively. The samples Z0, Z1 and Z3 are polycrystalline in nature, as revealed by the SAED pattern given as the inset of respective figures. The Z5 and Z7 samples are single crystalline in nature. **(f)** An example SAED pattern obtained from the Z7 sample. The double diffraction spots represented by the red line, in the single crystalline SAED pattern reveals the presence of twinning effect in the solid sheet samples.

**Figure 2:**
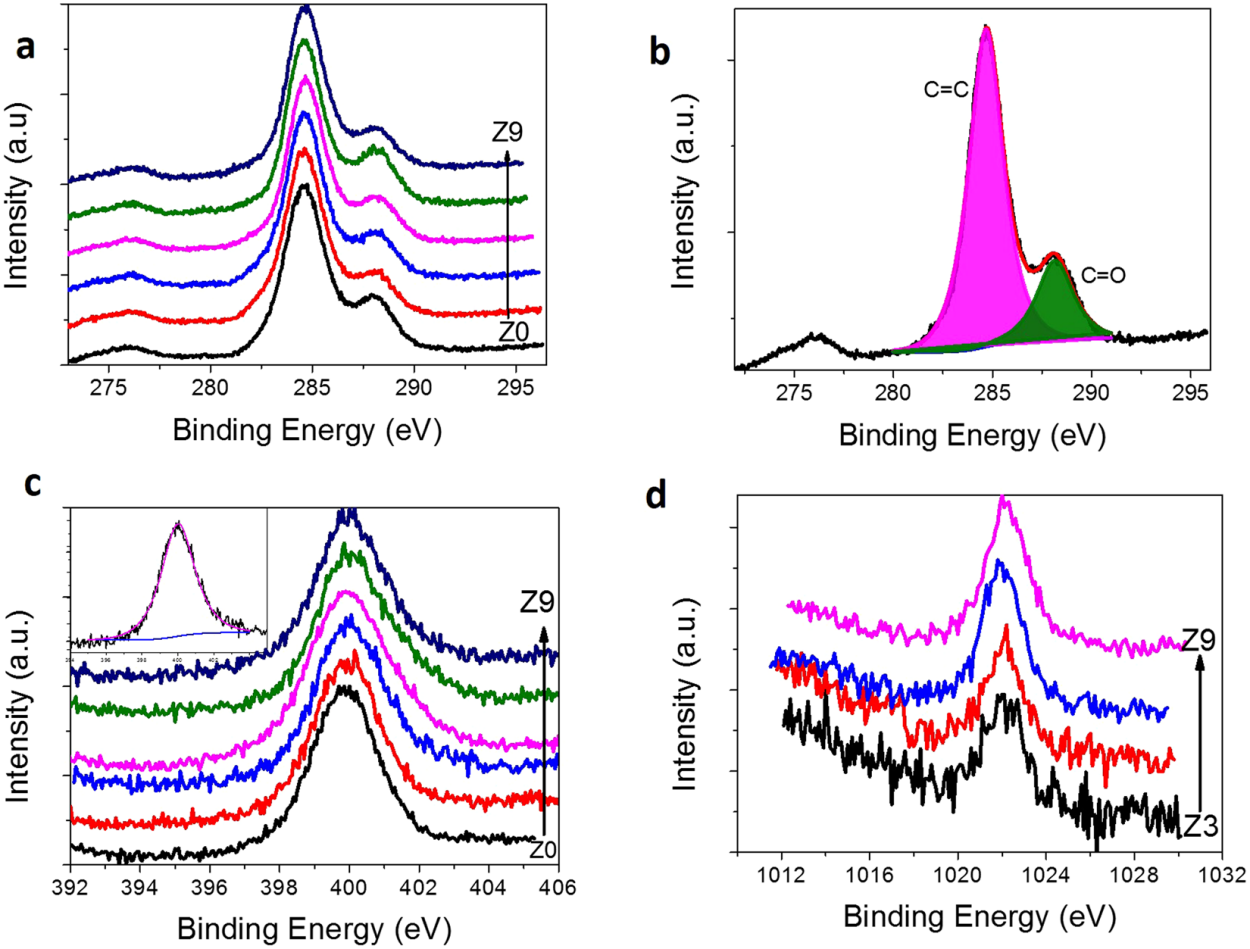
XPS analysis of GQDs and GQD solid sheet structures. (**a**) High resolution C1s spectra of GQDs and different sized solid sheets (Z0-Z9), (**b**) the deconvoluted C1s spectra as a representation, gives the evidence of graphitic C=C (at 284.6 eV) with less amount of carboxyl functionalities (at 288.1 eV), (**c**) N1s high resolution spectra showing the broad symmetric peak at 399.9 eV. It indicates that, the major contribution is from the graphitic nitrogen component. The inset shows the deconvoluted N1s spectra. (**d**) Zn2p high resolution spectra demonstrating the increase of Zn presence in the GQD solid sheets with increase in sheet size. XPS survey spectra and O1s spectra of all the as prepared samples are given in the Supplementary Fig. S2.

**Figure 3:**
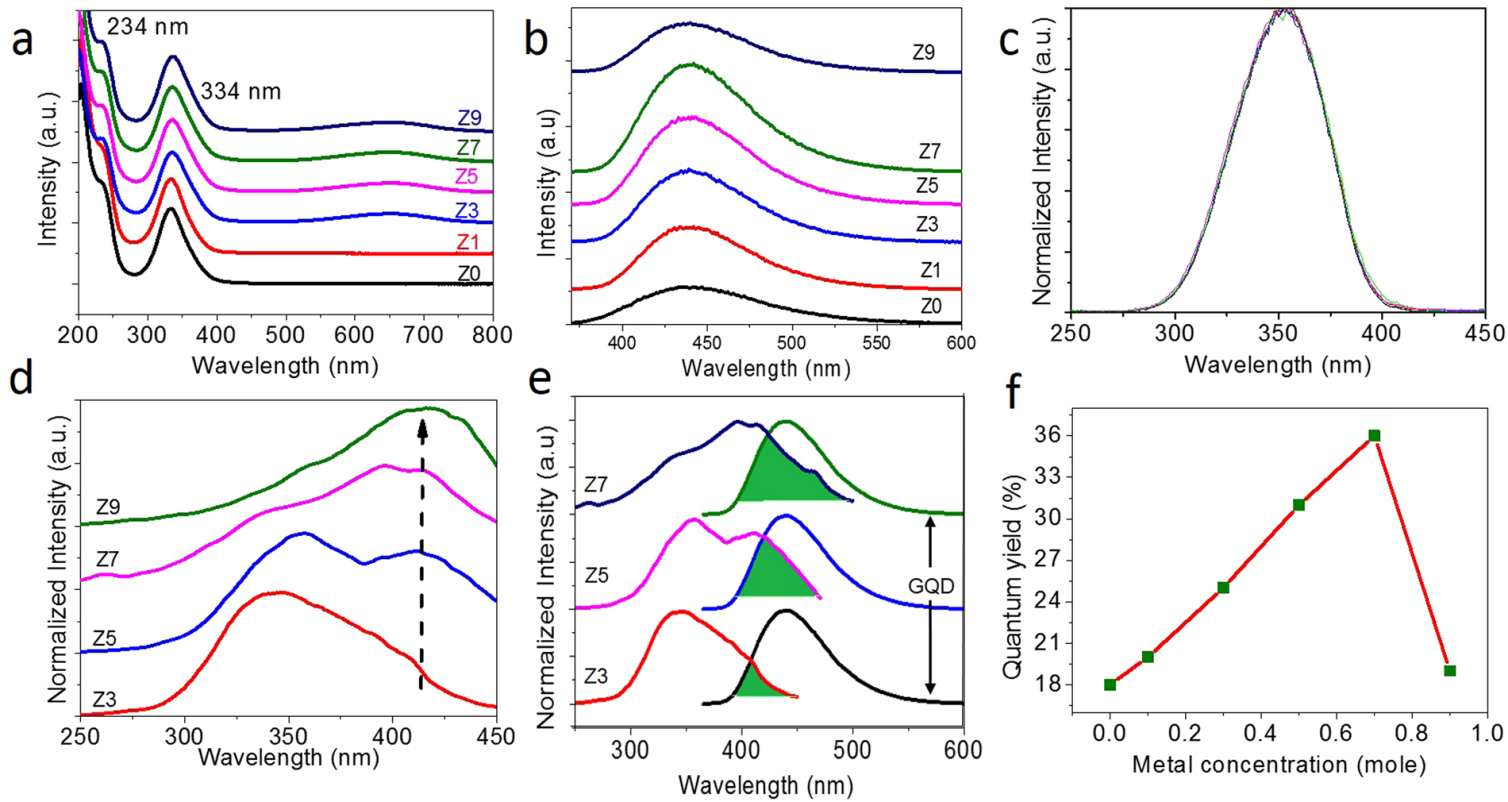
Optical properties of GQDs and GQD solid sheet structures. (**a**) UV-Vis absorption spectra of Z0-Z9 raw samples. It shows two absorption bands at 234 and 334 nm correspond to the π-π* (C=C) and n-π* (C=O/C=N) transitions, respectively, (**b**) emission spectra of raw samples showing increase in emission intensity upto Z7 and further a decrease for Z9 sample, (**c**) excitaion spectra of raw samples showing peak maxima at 350 nm, (**d**) excitation spectra of separated solid sheets show the shift in peak maxima from 350 to 410 nm for samples prepared with higher metal concentration, (**e**) spectral overlap between excitation of solid sheet samples [Z3, Z5 & Z7] and emission of GQD samples (**f**) A graph representing the quantum yield values as function of metal concentration in our samples. The excitation, emission, and quantum yield values are given as comparative table in the Supplementary Table S1.

Graphene samples were prepared by the hydrothermal method by taking citric acid, urea and zinc chloride as precursor molecules (further details are given in the methods). The as-prepared samples were then characterized in terms of morphology, chemical purity and absorption/emission properties. It has been observed that there is an increase in size of the graphene with increase in the zinc metal dopant concentration. Figure 1 shows the HR-TEM images of the as-obtained graphene samples prepared with different zinc metal concentrations: 0.0, 0.1, 0.3, 0.5 and 0.7 mole. In the absence of metal dopant, spherically shaped GQD morphology was obtained with an average size value of 5 nm (Z0 sample shown in Fig. 1a). The selective area electron diffraction (SAED) pattern corresponding to this sample depicts the polycrystalline nature (inset of Fig. 1a). TEM image and histogram showing the size distribution of GQDs are given in the Supplementary Fig. S1. In the metal-assisted synthesis, we noticed an increment in the average size of the graphene with increase in the metal concentration. For example, in the 0.1-mole metal-doped sample (Z1 sample shown in Fig. 1b), we obtained GQDs in the 10-nm size range that are polycrystalline in nature (inset of Fig. 1b). In the 0.3-mole doped sample (Z3 sample shown in Fig. 1c), the maximum size was increased further to approximately 100 nm with a polycrystalline nature (inset of Fig. 1c). Similarly, when the metal concentration was increased further to 0.5 (Z5 sample shown in Fig. 1d) and 0.7 mole (Z7 sample shown in Fig. 1f), we obtained well-grown micron-sized single-crystalline graphene sheets. It was further noted that all the raw samples prepared with metal doping contained GQDs as well, in the size range of 2–5 nm. The above results indicate that the average size of the graphene sheet increases with increase in the metal concentration. This is the first ever report from our research group on obtaining micron-sized graphene sheets by this simple bottom-up approach. The sample purity and structural quality of the as-prepared GQD and sheet samples were investigated by X-ray Photoelectron Spectroscopy (XPS) and X-ray Diffraction (XRD) analyses. From the XPS analysis, it was found that all the samples are more graphitic like, with a pronounced C=C peak at 284.6 eV^37^ (C1s spectra given in Fig. 2a). In addition, a weak peak at 288.0 eV was also observed, corresponding to the C=O groups present in the samples. The low oxygen-to-carbon ratio (0.23–0.28) is in accordance with good-quality graphene structures prepared by other methods^38^. It must be noted that the chemically derived graphene samples usually possess appreciable amounts of oxygen moieties, and their rarer presence in this case proves the quality of the as-prepared graphene samples by this method. Since all the samples were prepared at an optimized concentration of urea, there was no change in the intensity of the nitrogen peak in our samples. The nitrogen dopant percentage was approximately 10%. Furthermore, from the N1s XPS spectra (Fig. 2c), the broad symmetric peak tells us that the nitrogen exists mostly in graphitic form in all the samples. The Zn2p XPS spectra (Fig. 2d) corresponding to the zinc component at 1022.1 eV (Zn2p3/2) confirmed the presence of metal, and the peak intensity increased with the increase of the metal concentration in the sample. The calculated amounts of Zn metal present in our samples are 0.3, 0.4, 0.6 and 0.9% for the Z3, Z5, Z7 and Z9 samples, respectively. To confirm the structure of graphene, X-ray diffraction (Supplementary Fig. S3) was performed, and it revealed that the samples are structurally close to graphite, with an interlayer spacing (d spacing) value of 0.346 nm for the Z0, Z1 and Z3 samples. This d spacing value was slightly increased to 0.36 nm for the Z5, Z7 and Z9 samples, which can be attributed to the introduction of oxygen containing functional groups in between the layers of larger-sized sheet samples^39^. To obtain the optical properties of the raw samples, absorption and emission studies were carried out. Figure 3a and b show the absorption and emission spectra recorded from the raw samples (Z0-Z9). From the absorption spectra of the raw samples (Fig. 3a), we noticed an absorption band at 234 nm due to the π-π* transition of the C=C bond present in the graphene. Similarly, an absorption band at 334 nm was also noticed due to the n-π* transition of the C=O/C=N bonds present in the sample^9, 29^. The photoluminescence property of the raw samples was analysed using a spectrofluorimeter instrument. The quantum yield values were determined by comparing our samples’ emissions with the emission from the quinine sulphate dye reference. Interestingly, the results showed an increasing trend in the emission yield with the increase of graphene size. The Z0 GQD sample has shown an emission yield value of 18% at 440 nm (the GQD sample exhibited excitation energy independent emission behaviour as shown in Supplementary Fig. S4). Samples Z1, Z3, Z5 and Z7 showed increasing trends in emission yield in the range from 20 to 36% with the increase of the average sheet size. In the case of GQDs, it has been reported that size, edge and doping (nitrogen) effects^9, 20, 24^ are the major contributing factors for blue emission and we also attribute the same reasons for the emissions from our GQDs. In other samples, the observed increases in the emission yields have indicated that there is a co-relation between the increasing trends of the average size and emission yield: the larger the sheet size, the stronger the emission yield. The ultimately obtained strong blue emission from the sheet sample is an unusual result in graphene research, and therefore, one must understand the scientific reason for this new emission behaviour. To answer this question, we have subjected the as-prepared raw samples to a filtering process so that the possible presence of graphene QDs and their contribution to the emission yield can be excluded. In this way, we obtained two sets of samples with different emission yield values from all the raw samples. Before measuring the quantum yield values, the optical absorption, excitation and emission spectra were recorded from the separated samples. For example, the Z7 coded sample, which was filtered out of the membrane, gave 18% emission yield at 440 nm. On the other hand, the sample that was left out in the membrane gave a poor yield of approximately 3% at 440 nm, making us surprise over the missing of the original maximum emission yield value of 36% from the corresponding raw sample. To understand the reason for this discrepancy, we analysed the size and shape details of the samples. The filtered-out samples are in the size range of 2–5 nm and they are GQDs. On the other hand, the filtered-in precipitates have sheet morphology. The observed results have therefore indicated that the larger-sized graphene sheets are poor in emission when they are separated out of GQDs. At the same time, the filtered-out GQD sample is able to produce emissions similar to those of the Z0 raw sample with a yield of 18%. Surprisingly, an increased emission yield value of approximately 36% was obtained when we mixed the filtered-out and filtered-in samples together, which is similar to the value of the raw sample. A similar filtering process was also extended to other samples, and in all cases, we obtained 18% yield from the filtered-out GQD samples at 440 nm because of their size similarity with the Z0 raw sample. In all cases, the remixing process resulted in the regaining of emission yield values similar to the corresponding raw samples’ emission yields. The observed experimental results indicate that there is an interaction between GQDs and sheet morphology that leads to a higher emission yield of more than 18%. We believe that this interaction could be a Forster Resonance Energy Transfer (FRET) type, in which energy from the excited donor is non-radiatively transferred to the acceptor. The fundamental conditions for the FRET-based interaction are (i) spectral overlap between the donor emission and acceptor absorption/excitation and (ii) minimum distance between the donor and acceptor, which directly controls the dipole-dipole interactions^40^. The other factors that affect the FRET efficiency are the screening of the electric field of the donor dipole in the acceptor medium and the dimensionalities of the donor and acceptor^41^. To ensure that FRET occurs in the as-prepared samples, we obtained the excitation spectra from the filtered-out GQD and filtered-in sheet samples separately. Interestingly, we noticed a new excitation peak at 410 nm from the sheet sample (Fig. 3d), which is red-shifted with respect to the excitation peak of the filtered GQD sample (350 nm). This excitation peak is not visible in the raw sample because of the dominant absorption of the GQDs. This peak weakly appears for the Z3 sample, and its intensity increases with increasing concentration of metal dopant, as indicated in Fig. 3d. (We have not included the excitation spectra of the filtered-in Z1 sample. This is because, due to the size similarity with the Z0 quantum dot sample, the excitation band is similar to that of the Z0 sample with a maximum peak at 350 nm.) When this new excitation band of the graphene solid sheet at 410 nm was plotted together with the emission spectra of the graphene quantum dots, there appeared the overlapping of bands at the higher-energy side of the emission spectrum, as shown in Fig. 3e. The percentage of overlap between the excitation spectra of the sheet sample and emission spectra of the GQD sample shows an increasing trend with the increase in the sheet size, and as this trend is in accordance with the increasing emission trend, we strongly believe that there is an energy transfer interaction between the GQD and the sheet sample. The GQD acts as the donor and the sheet acts as an acceptor, which satisfies one of the conditions for the energy transfer interaction. The other important condition for the energy transfer interaction is a minimum non-contact distance between the two interacting systems. This naturally exists between them because the surface-attached functional groups in both the sheet and dot samples keep them slightly away from direct contact. In general, reports say that the interaction between a graphene sheet and other QD systems is usually of an emission quenching type, followed by the transfer of electrons from QDs to graphene. However, in contrast to the previous observations, we have noticed an emission-producing graphene sheet sample via the energy transfer process. In the reported cases, the graphene sheet behaved like a metal and therefore quenched the emission from the interacting QD^42^. Here the band gap seems to be induced in graphene sheets, and as a consequence, an emission-producing-type energy transfer interaction results. It is well known that whenever the two interacting systems have a band gap and the conditions for energy-transfer-type interaction are satisfied, emissions are released from the acceptor system, as reported elsewhere^33, 43^. In those reported cases, both the donor and acceptor are CdSe QD systems, and the energy is resonantly transferred from the smaller-sized QDs to the larger-sized QDs. Reports are also available that demonstrate FRET between QD donors and biomolecule/dye acceptors. For example, the emission-producing-type FRET between ZnS QD and β-carotene was demonstrated. In this case, a spectral overlap occurred between the defect emission bands of ZnS QDs and the absorption band of β-carotene, leading to emissions from β-carotene at 600 nm^44^. Here, the graphene sheet, being an acceptor system with its band-gap-opened state, produces the maximum emission yield. Recall that if the interconnection process has resulted in metallic graphene, then it is not possible to induce emissions. Therefore, the as-prepared graphene sheets are named graphene quantum dot solid sheets. We also prepared a graphene sheet sample with a higher zinc content of 0.9 mole to understand the nature of the growth and emission trends. Surprisingly, the emission yield from this raw sample is only 18%. To understand this decrease in emissions, the raw sample was subjected to a filtering process to obtain dot and sheet samples, and then they were subjected to HR-TEM and emission studies, as performed previously on the other samples. Additionally, the filtered-out GQD sample showed a usual emission yield value of 18%. Similarly, the filtered-in graphene sheet sample produced very poor yield, as usual, when it was excited directly. However, when the samples were excited together, surprisingly, there was no strong emission, as in the case of previous samples, even though there was a better overlap between the emissions and excitations bands of the dot and sheet samples (the spectral matching graph is given in Supplementary Fig. S5). The HR-TEM investigations showed (Supplementary Fig. S6) that the sheet samples were very thick, and thus the energy transfer interaction was not favoured. The thickness-dependent energy transfer interaction was studied with graphene and CdSe/CdZnS QDs through coupling the quantum dots with graphene-layer films on a glass substrate by A. Raja *et al*.^41^. The reported critical thickness of the graphene layer, up to which the energy transfer interaction is maximum, is approximately 5 layers (3–4 nm) according to their electron lifetime values. In the present case, an increased energy transfer interaction that resulted in maximum emission yield was observed up to sample Z7, which has a thickness value of 8 nm, as measured from the AFM analysis (the other sample thickness values are 4 nm for GQDs and 5 nm for samples Z3 and Z5, according to the AFM analysis given in Supplementary Fig. S7). The thickness value of our GQD solid-sheet sample at which an effective energy transfer interaction was observed, is relatively high. Since we have observed the FRET mechanism in the solution form of the sample, GQDs can interact with solid-sheet samples on either side of the sheet, and therefore, there is a chance for effective interaction (of the energy transfer type) above the reported minimum critical thickness value (5 layers/3–4 nm).

**Figure 4:**
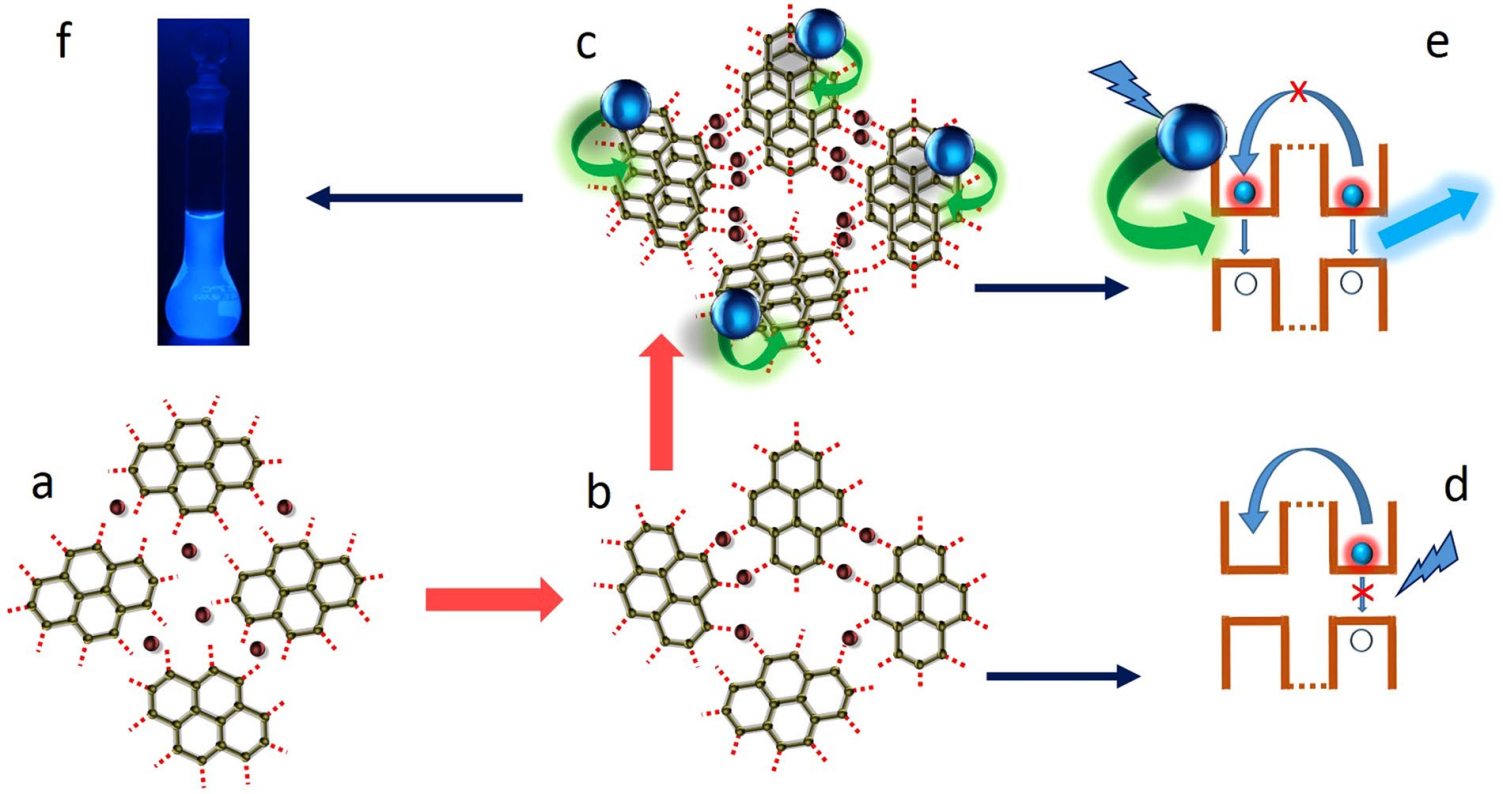
Schematic representation of the proposed growth process. (**a**,**b**) Graphene quantum dots (GQDs) are interconnected through binding with metal atoms, (**c**) stacking of interconnected graphene solid sheet layers and indirect FRET excitation by the GQDs emission, (**d**) quantum well design of the sheet sample, representing the non radiative relaxation of photoexcited electrons, when excited directly. This is due to the delocalization of photoexcited e-h pairs. (**e**) In the solid sheet - GQDs coupled structure, the photoexcited electrons present in all the excited states because of the simultaneous excitation of all the domains by FRET process and therefore the delocalization process is suppressed, (**f**) Photographic image of Z7 sample when illuminated with UV light. The CIE chromaticity diagram representing the emission from Z7 sample is given in the Supplementary Fig. S8.

Now, the following question arises: why did the sheet samples not produce high emission yields when excited directly at 350 nm? The answer is that the sheet samples have poor absorption at 350 nm. Interestingly, these sheet samples do not produce strong emission, even when excited at 410 nm. We believe that the reason could be related to the nature of the GQD solid sheet structure: the GQDs are connected here, and therefore, the photoexcited electron-hole (e-h) pair will preferably relax through these coupled structures, finally undergoing a non-radiative recombination through the mid-band-gap energy levels that arise due to the presence of surface-attached functional groups in the solid sheet structure. The lifetime measurements, which will be discussed later, also support this claim. However, in the presence of GQDs, most of the domains in the graphene sheets are excited simultaneously through an energy-transfer-assisted interaction with GQDs, and therefore, the excited electrons are in each of the excited states of the respective domains, as schematically depicted in Fig. 4e. In this condition, electron/hole diffusion from one domain to another is blocked, and thus the excited electrons preferably recombine with the holes in the valence band edge states of the respective domains, producing a bright PL emission. It is thought that the appearance of the excitation peak at 410 nm from the sheet samples is the main reason for the emission maximum and associated energy transfer interaction. Then, the question arises as to the origin of this new excitation band from the sheet sample. We strongly believe that this new excitation band at 410 nm is associated with the quantum size effect of the sp^2^ domains in the solid sheets. This band is not visible in GQDs, which could be because the dominant contribution to the excitation spectrum is from the surface-attached functional groups that usually show a band at 350 nm. Since the surface-to-volume ratio is very high in the GQDs, the contribution of surface-attached functional groups to the absorption/excitation spectra will be high. In the case of GQD solid sheets, the surface-to-volume ratio is minimum, which means that the surface effect is minimum, and therefore, the quantum-size-effect-induced excitation band starts to appear. It is because of this effect in the solid sheets the emission is Stokes-shifted by only 30 nm.

The time-resolved photoluminescence behaviour of the FRET donor system is usually monitored in both the presence and absence of the acceptor system, and the difference in the average lifetime values will be used to calculate the energy transfer rate from donor to acceptor. Well-separated donor and acceptor emissions are required for the effective monitoring of lifetime values. In our present work, since our donor and acceptor emissions are at the same wavelength, it is difficult to measure the lifetime values of the donor system (in the donor emission position) in the absence and presence of the acceptor system. Therefore, we have monitored the PL decay curve of the acceptor system at 440 nm and compared it with the PL decay curve of the donor-acceptor mixture system to confirm the energy transfer mechanism. It has been reported that the average lifetime value of the mixture system is usually higher than the average lifetime value of the acceptor system because the charge carriers that are photoexcited in the donor system are non-radiatively transferred to the acceptor system and excite the acceptor system to produce emission; therefore, naturally, a longer lifetime value results^45^. In our work, we have obtained an average lifetime value of 7.85 ns from the mixture sample, which is higher than the acceptor systems’ average lifetime value of 6.03 ns (obtained from the PL decay curves shown in Supplementary Fig. S9a). The observed results have thus confirmed the energy transfer from donor to acceptor. The lifetime decay is faster in the sheet-only sample than in the mixed sample. This observation supports our claim that the photoexcited electrons dissociate faster in the sheet samples and are slowed down when mixed with the GQDs.

We have also conducted pH-sensitive emission measurements (Supplementary Fig. S10) to confirm the intrinsic nature of the emission from GQD solid sheets. It is well documented that the emission that originates from carbene states and other surface states is usually quenched at low pH values (pH = 1)^20^. In our GQDs, the emission is also quenched, leaving behind a small amount of emission at low pH value. This minimum unaffected emission from the GQDs originates from the quantum size effect. However, in the case of GQD solid sheets, the emission is insensitive to pH, which indicates that the emission of the solid-sheet sample is only due to the size effect [excitation dependent emission analysis of solid sheet sample is given in supplementary Fig. S11]. Our raw samples also showed a sensitivity to pH because of the pH-sensitive GQD donor, which induced strong energy-transfer-assisted emission from the raw samples. Hence, to induce strong pH-insensitive emission from the sheet sample, the pH-independent emission-producing ZnS QD system has been used as the donor. The defect-related emission from ZnS QDs (which have maximum emission at 400 nm) served as the donor for the energy transfer process, which induced pH-insensitive strong blue emission at 440 nm from the solid-sheet sample with an emission yield of 20%. Since donor and acceptor emissions are well separated in the graphene solid-sheet-ZnS QD combination, we have recorded emission decay curves (Supplementary Fig. S9b) from the donor alone and the donor-acceptor mixture samples (at the donor emission maximum of 400 nm), and we have calculated the energy transfer rate as 10% from the average lifetime values. This result confirms the pH insensitivity of the emission from the solid-sheet samples, which is a true intrinsic effect.

**Figure 5:**
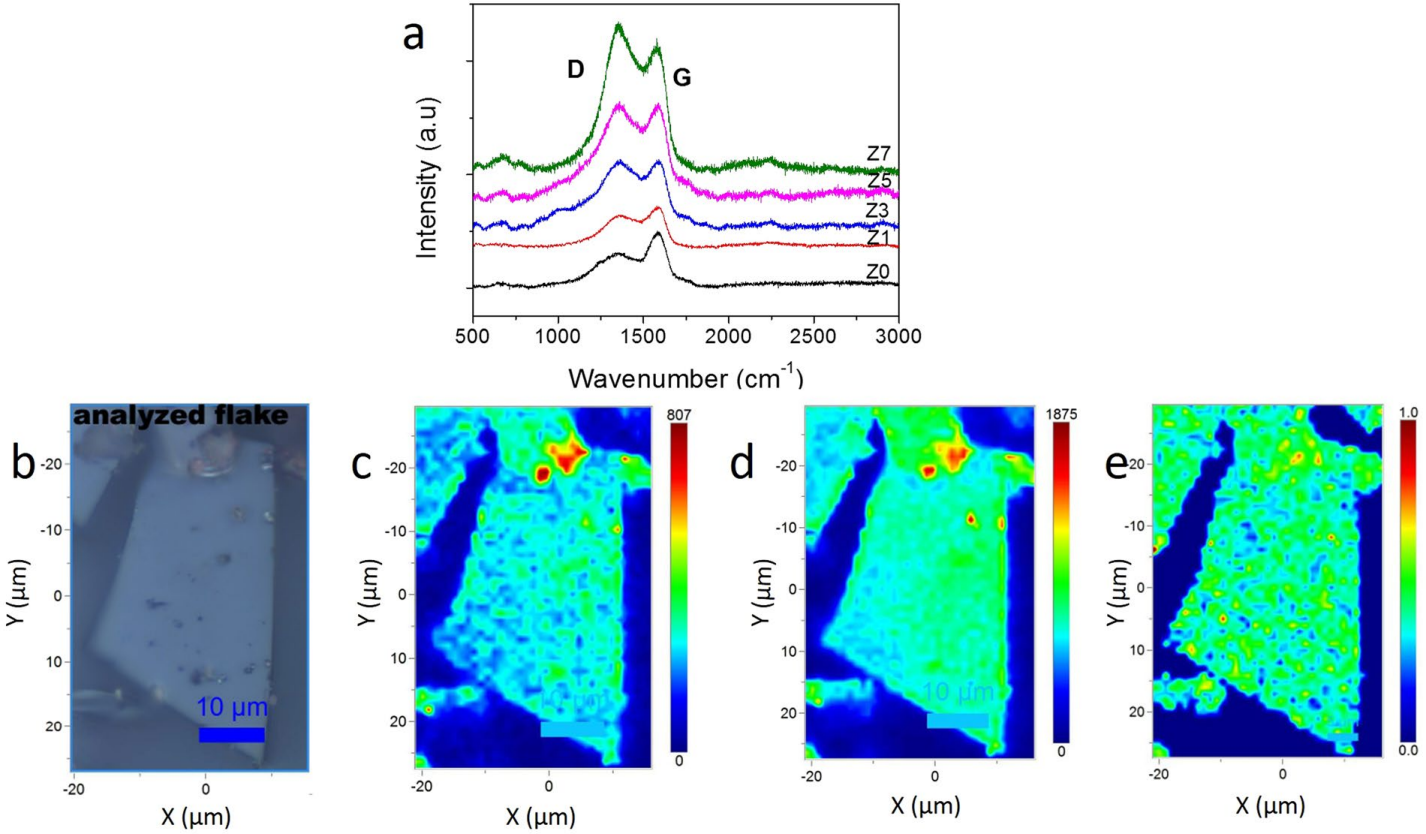
Raman analysis of GQDs and GQD solid sheet structures. (**a**) Raman spectra of the raw samples (Z0-Z7), showing D and G bands. The D band increases with the increase of metal concentration. (**b**) Optical microscope image of a GQD interconnected solid sheet (Z7), (**c**) Raman mapping of D band, (**d**) Raman mapping of G band, (**e**) area ratio of D band to G band. It clearly shows the distribution G and D bands in whole area of the solid sheet.

All of these experimental results have indicated that the metal atom plays an important role in interconnecting the GQDs into sheet-like structures, without affecting the quantum behaviour of the graphene dots. By simply prolonging the growth duration, it was not possible to increase the average size of the GQD (Z0 sample) above a few tens of nanometres. This means that it is not possible to grow micron-sized sheet samples by simply extending the growth duration by this method. The metal atom presence helped to grow the graphene structure from tens of nanometres to a few micrometres. The dopant zinc metal atom seems to bind to the edges of two GQDs, connecting them together into a sheet form. In this interconnection process, the zinc metal atom cannot form a perfect sp^2^ carbon network because of the difference in the valence of the metal atom. As a consequence, an incomplete carbon network results, from which the GQD solid sheets are formed. When the metal content increases, the lateral dimension of the sheet also increases through the zinc-metal-atom-assisted interconnection method, thus converting the GQDs into an interconnected micron-sized solid sheet (Fig. 4 schematically represents the GQD interconnection process through metal atoms to form solid sheets). When the size is increased, it can be a platform for the nucleation and growth of other GQDs, and as a result, the thickness also increases in parallel. The obtained solid sheets are unique in their structure and properties compared with the other reported graphene sheet systems obtained by other preparation methods. The proposed growth mechanism was confirmed by HR-TEM, Raman and Atomic Force Microscopy experimental analyses. From the HR-TEM analysis, a closer look at the SAED pattern of the single-crystalline samples (insets of Fig. 1d and f), such as Z5 and Z7, has revealed double diffraction spots; this is due to the twinning effect in the graphene solid sheet. Twin boundaries usually occur when two crystals of the same type inter-grow with a slight orientation mismatch between them. Crystal twinning also occurs when atoms or molecules attach erroneously in such a way that two crystals grow out of or into each other^46^. Here, the zinc metal atom binds to the edges of individual GQDs, which already have the twisting defect, to grow into a micron-sized structure; while forming such larger structures, there is always a higher chance of a slight orientation mismatch between the interconnecting dots, leading to the twinning effect in the graphene solid sheets (the HR-TEM image showing the presence of moire pattern in the Z7 sample is given in the Supplementary Fig. S12). The existence of the twining effect in the sheet samples could be therefore considered as evidence for the bottom-up growth of the sheets. Raman analysis (Fig. 5) has shown D and G bands at 1350 cm^−1^ and 1580 cm^−1^, respectively^17^, for all of the raw samples. The Raman spectra of samples Z0 and Z1 have shown a G-band-dominated structure, which indicates that the as-grown graphene quantum dots are more graphene-like. The spectra corresponding to samples Z3-Z7 have D-band dominance, meaning that an sp^3^-type disorder is introduced into the network due to the metal doping effect. As the metal content increases, the intensity of the D band starts increasing because of the increase in the structural disorder. It should be noted that the unsaturated carbon atoms in the network form an sp^3^-like disorder with oxygen atoms, which in turn results in the enhancement of the D band with the increase of the solid-sheet dimension. We did not notice the 2D band from the GQD solid sheets when it was measured from the raw sample. However, the filtered-in solid-sheet sample showed a 2D band at 2800 cm^−1^ (supplementary Fig. S13), confirming the formation of a graphene sheet. In the raw samples, we could not observe the 2D band due to the dominant contribution of GQDs, which mostly produce D and G bands. Further, Raman analysis also confirmed the atomic zinc doping by not showing any shift in the G-band position of the metal-doped samples. This result indicates that the zinc metal is not in cluster form; instead, it is distributed throughout the graphene network as atomic zinc. Atomic doping of graphene is mostly a theoretically studied topic by researchers. Only a few reports discuss the incorporation of metal atoms into the graphene network, mostly with transition metal atoms, such as Ni, Mn, Fe, and Co. The calculations revealed that these transition-metal atoms are preferably occupying the hollow sites and vacancies created by the pyridinic/pyrrolic defects on the graphene^47–50^. Ushiro *et al*. reported the trapping of Ni atoms at graphene edges through EXAFS studies^51^. This is also true in our case: the metal atom preferably binds to the edge site and the same metal is involved in interconnecting the GQDs, thus helping to grow large-sized graphene sheets.

### X-ray absorption spectroscopy of GQDs and solid sheets

Since our samples are GQD solid sheets interconnected by Zn metal atoms, experimental techniques such as X-ray diffraction cannot provide information related to the nature of Zn in our samples. Hence, X-ray absorption spectroscopy, which is widely used to probe the local bonding structures of materials, was used here to acquire information about the metal binding with GQDs in our solid-sheet samples.

**Figure 6:**
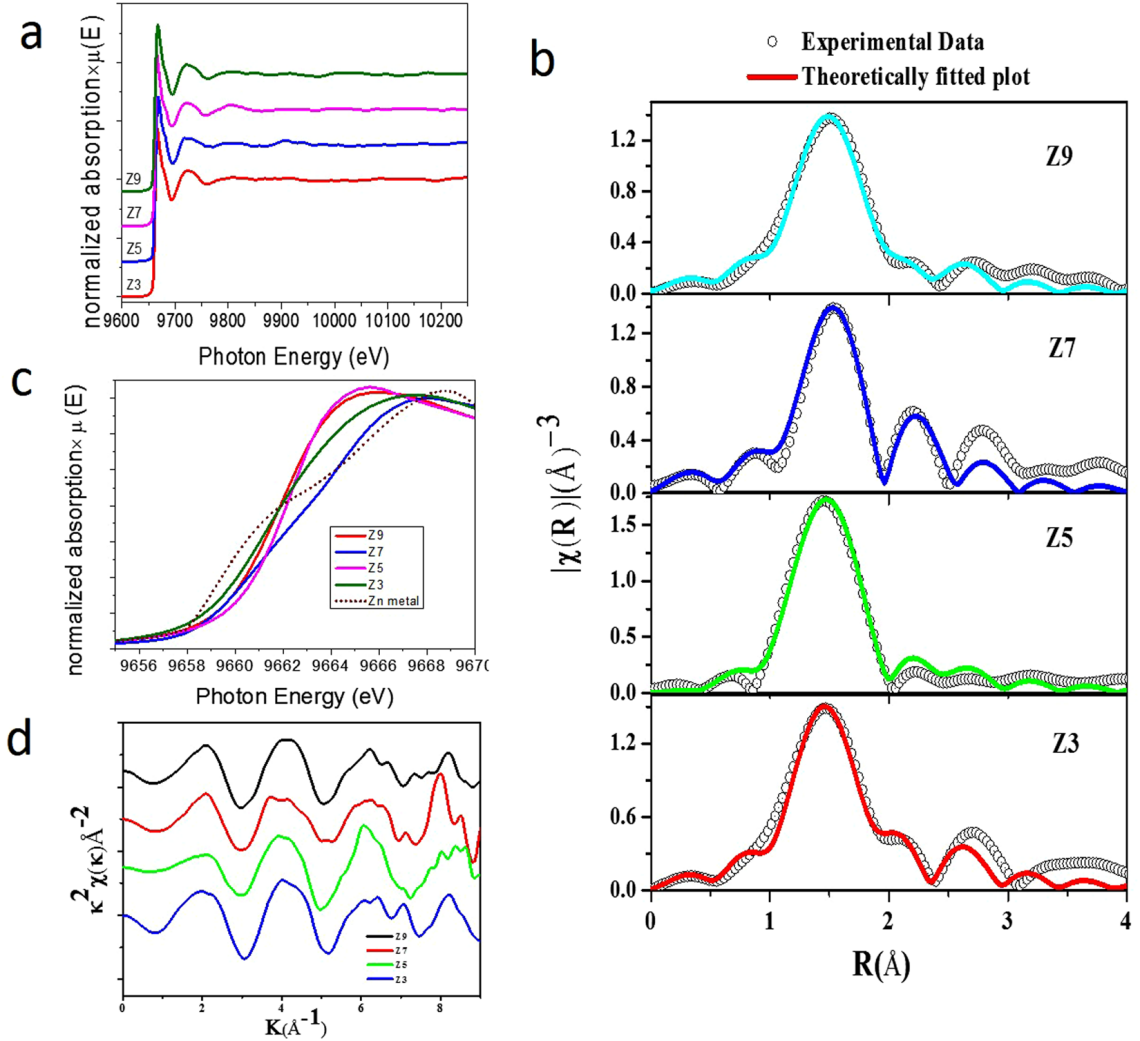
X-Ray absorption spectroscopy measurements of GQD solid sheet samples at Zn *K*-edge. (**a**) EXAFS spectra at Zn K-edge of solid sheet samples, (**b**) experimental χ(R) vs R data fitted with the theoretically generated plot at Zn k-edge, (**c**) enlarged absorption edge [near edge] of Zn K-edge spectra showing the higher valence state of Zn in our samples compared to that of metallic Zn, (**d**) normalized *k* ^2^ weighted χ(*k*) spectra of the GQD solid sheets at the Zn k-edge.

The EXAFS measurements at the Zn K-edge were carried out using a hard X-ray beam from the BL-9 beamline at the Indus-2 synchrotron radiation source at RRCAT, Indore, India. The EXAFS spectra (Fig. 6a and c) of solid sheets interconnected by Zn metal atoms reveal the higher valence state of Zn in our samples compared to metallic Zn. This is due to the binding of electropositive Zn atoms with electronegative C and O atoms. A shift in the absorption edge towards the higher-energy side was observed in our solid-sheet samples compared to metallic Zn, and moreover, the shift is predominantly higher for the 0.7-mole Zn-doped sample. This indicates the increased electropositive nature of Zn in the Z7 sample, which results in a slight decrease in the ionic radius of Zn and a corresponding increase in the bond lengths around the Zn atom. To better understand the local bonding of the Zn atoms in our solid-sheet samples, a theoretically generated model was used along with the experimental data. Bond distances obtained from the optimized structure shown in Fig. 9c (to be discussed later) were used to generate the theoretical scattering paths. The *k* ^2^- weighted *χ*(*k*) spectrum depicted in Fig. 6d shows uniform, well-defined and undamped oscillations within the range up to 8.5 Å^−1^, which is quite good for fitting of the experimental data up to 3 Å. This shows that the degree of disorder defined by the Debye-Waller factor is not high. The experimental data were in accordance with the theoretically generated plots shown in Fig. 6b. In the *χ*(*R*) vs *R* plot, the major peak that appears at ~1.5 Å is due to the mixed contributions of the back-scattering wave from the nearby four C atoms and one O atom situated at distances of 1.91 Å and 2.10 Å in the first and second co-ordination shells. Another peak with a smaller amplitude appears at ~2.70 Å due to the third coordination shell of Zn atoms at 2.77 Å. The best fitting parameters obtained from the EXAFS data analysis are summarized in Supplementary Table S2. The obtained results show that the bond distances of the sample with higher Zn content (Z7) were increased. Furthermore, the sample prepared with 0.9 mole Zn behaved quite differently, showing a shift in the lower-energy side. This means that there was a decrease in the bond distances, which could be because of the presence of precipitated Zn atoms in the graphene solid-sheet sample (Z9). The overall observed experimental results from the X-ray absorption studies reveal that the metal atom helps to interconnect the graphene nanodomains into larger solid sheets. In addition, no metallic cluster formation is observed, and our elemental mapping analysis (Supplementary Fig. S14) also confirms the distribution of atomic zinc in our solid-sheet samples. The other important point to be noted is that the Zn atom is coordinated with both C and O atoms.

**Figure 7:**
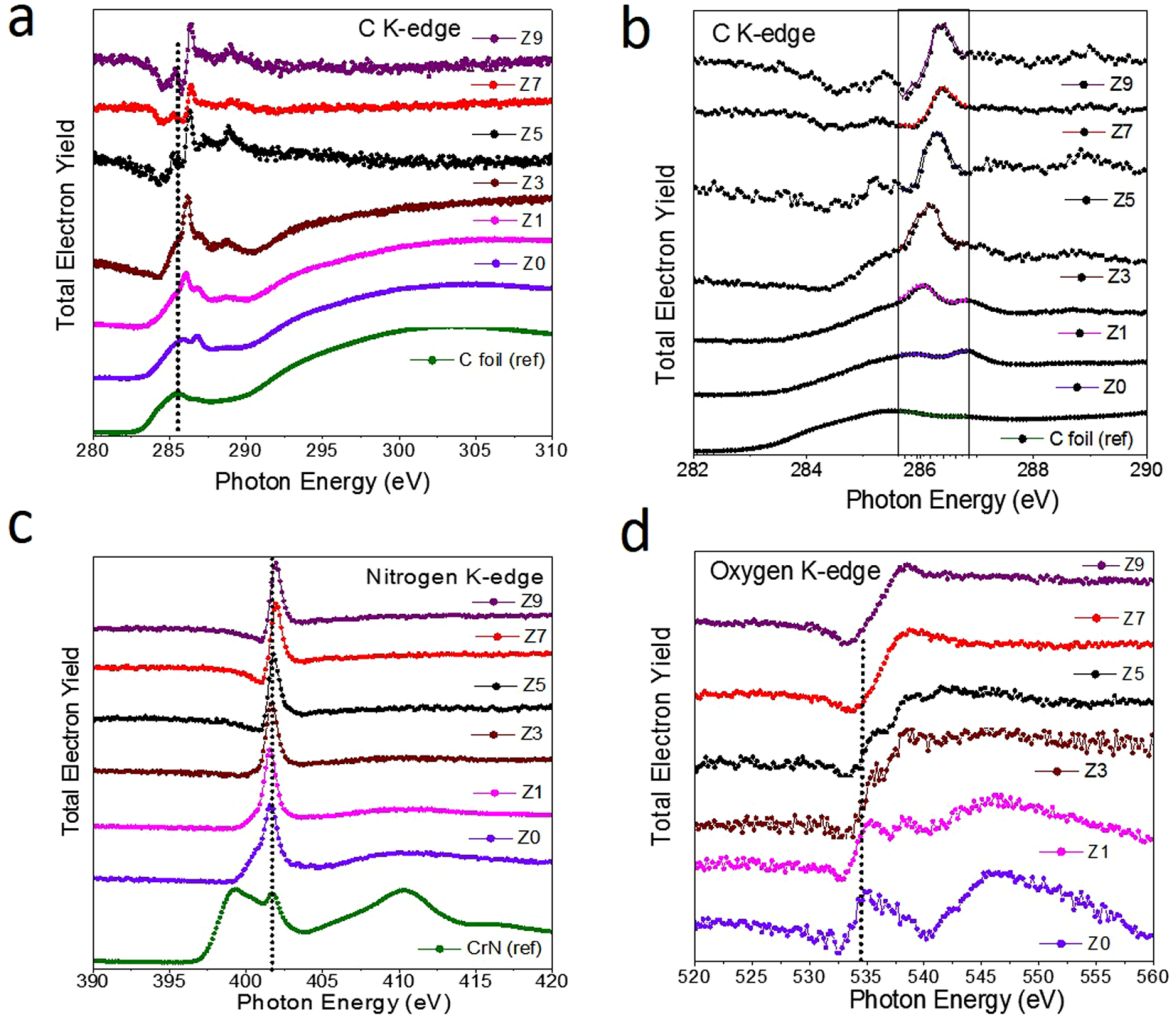
X-Ray absorption spectroscopy measurements of GQDs and GQD solid sheet structures at C, N and O *K*-edges. (**a**) C *K*-edge spectra of GQDs and solid sheet samples (Z0-Z9), (**b**) enlarged C-*K* edge spectra showing the rise of excitonic peak at 286.3 eV with metal doping effect, (**c**) N *K*-edge showing the presence of graphitic nitrogen with a sharp peak at 401.1 eV, (**d**) O K-edge spectra showing the presence of C-O and C=O in our samples.

The elemental composition obtained from the XPS analysis showed the presence of C, O and N along with Zn in our samples. Based on these XPS results, X-ray absorption was also measured at the C, N and O *K*-edges. The C *K*-edge, N *K*-edge and O *K*-edge spectra were measured using soft X-rays from the BL-1 beamline at the Indus-2 synchrotron radiation source at RRCAT, Indore, India^52^. The experimental conditions are provided in the Methods section. The C *K*-edge spectra of the GQDs and solid-sheet samples were measured in the energy range of 280–310 eV (Fig. 7a). They showed the characteristic peaks at 285.3, 287.3 and 288.7 eV corresponding to 1s-π* transitions of C=C, C-O-C, and C=O^53, 54^, respectively. In addition to these peaks, an interesting new peak began to appear at 286.3 eV from the Z1 (0.1 mole Zn) sample, and its intensity increased with the increase of the GQD solid-sheet dimension (Fig. 7b). We believe that this sharp peak is an excitonic peak due to the quantum confinement effect in the solid-sheet sample. The GQDs that build up the solid sheet retain their quantum behaviour in the sheet morphology as well, and therefore, the excitonic peak is made visible in the solid sheet sample. When the sheet dimension increases, the concentration of GQDs naturally increases, and therefore, a rise in the peak intensity results. A similar excitonic peak was also observed from the graphene oxide sample prepared by the Bordie’s method^53^. In the reported case, the presence of isolated nanoscale sp^2^ domains is responsible for the excitonic peak at 287 eV. Similarly, the N *K*-edge and O *K*-edge spectra were also measured at the energy ranges of 400 and 530 eV, respectively. The N *K*-edge spectrum (Fig. 7c) showed a single peak at 401.5 eV, which was attributed to the 1s-π* transition of graphitic nitrogen, as reported elsewhere^55^. This observation is analogous to our XPS N1s spectra, which showed a symmetric peak corresponding to the dominant graphitic nitrogen in the GQD solid-sheet samples. In the O *K*-edge spectra (Fig. 7d), a peak at 534.2 eV corresponding to the 1s-π* transition of C-O^56^ and a broad peak beyond 540 eV due to the superposition of 1s-σ* transitions^57^ of C-O, O-H, and C-O-C were observed for the GQD sample. The C-O peak showed a slight blue-shift for the Z1 sample upon metal doping, and the shift increased further in the Z3 and Z5 samples. This peak is not visible in the Z7 and Z9 samples due to the predominant appearance of the C=O peak, as can be seen in the XAS spectra. This observation indicates that the C-O in the graphene network is possibly in local coordination with other nearest-neighbour (metal) atoms in the solid sheets, resulting in a noticeable shift in the peak position towards the higher-energy side. For the 0.1-mole Zn-doped sample (Z1), a peak at 538.2 eV, which is related to the 1s-σ* transition of C=O^54^, starts to appear. This C=O peak is not well resolved in the GQD sample and becomes more visible for the higher-metal-content samples. The peak intensity increases with the increase of sheet dimension, without showing any shift in its peak position. This means that the C=O bond is intact, and thus there is no shift in the peak position. Its presence/concentration is increasing with the increase in the sheet dimension. We believe that the increased presence of oxygen containing functional groups increases the structural stability of the graphene QD solid sheet.

### DFT-based quantum chemical calculations

**Figure 8:**
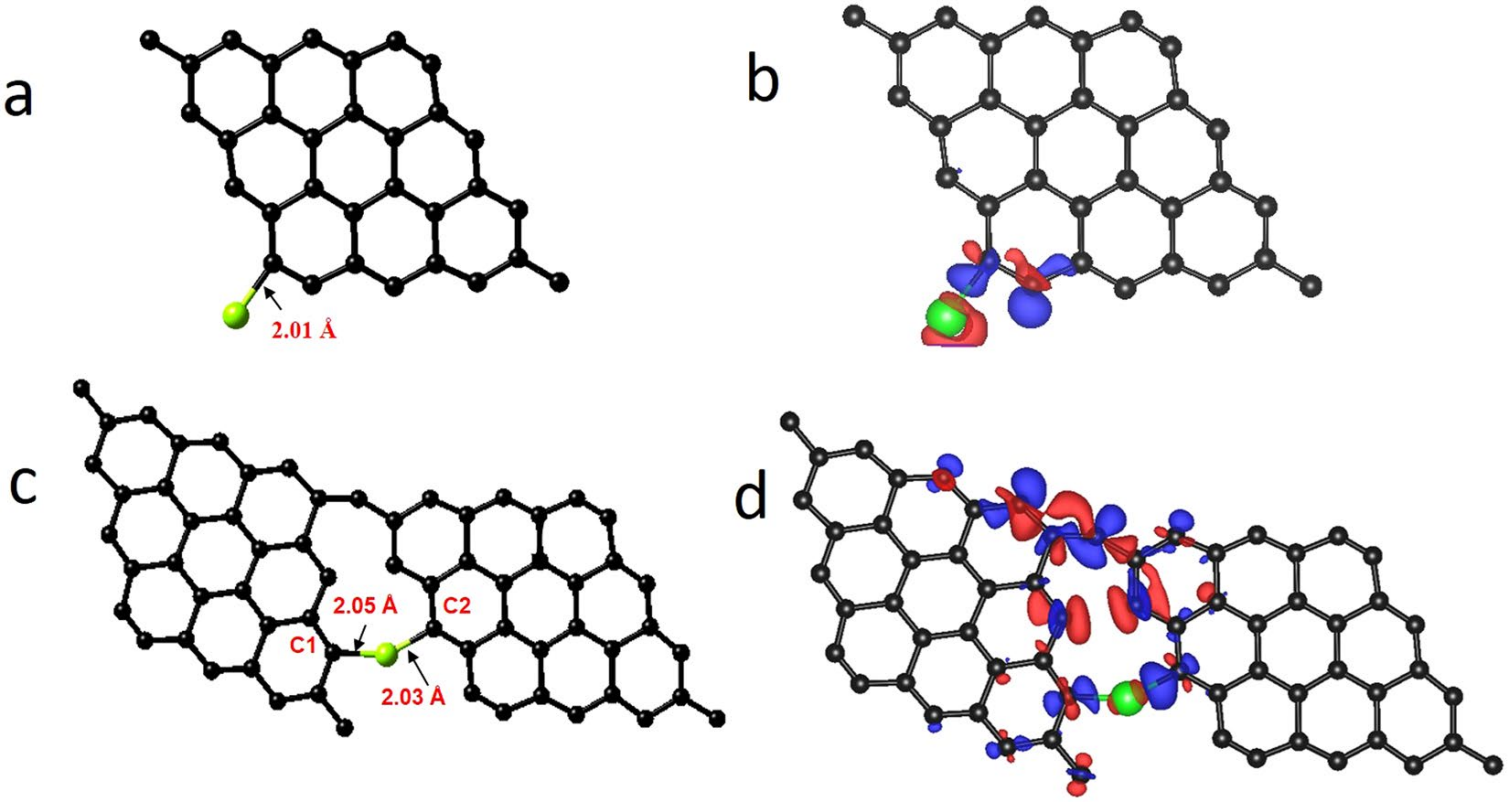
Metal atom incorporated Graphene structure models used for DFT and Bader charge calculations. (**a**) Graphene sheet interacting with Zn atom at the edge site, (**b**) electron density difference plot of Zn-edge interacting graphene sheet, (**c**) graphene sheets interconnected through zinc atom, (**d**) electron density difference plots of graphene sheets interconnected with zinc atom. It provides the theoretical evidence for the interconnection process and the electron density difference plots show the sharing of charges in the interconnection process.

Since experimental observations have confirmed the role of zinc metal atoms in the growth of graphene nanostructures, electronic structure calculations were performed (based on the DFT method) to confirm the role of zinc metal atoms in the growth of graphene. A graphene sheet containing 32 carbon atoms (as 9 benzene rings) was constructed, and zinc metal atoms were allowed to interact with them at different sites (Supplementary Fig. S16). This includes three sites on the surface of the graphene sheets (namely, the top site, bridge site and hollow site) and one edge site (Fig. 8a), and adsorption energies were calculated. From the calculations, it was found that, of the three surface sites, the hollow site was the most interactive site for the Zn atoms, with a corresponding adsorption energy value of −0.15 eV and a distance of 3.41 Å between them. The adsorption energy value for the top site and bridge site zinc atom is −0.13 eV and the distances between the Zn atom and graphene layer interacting at the top site and the bridge site are 3.46 Å and 3.37 Å, respectively. A similar calculation was performed for a Zn-attached graphene sheet (edge site), and the calculated adsorption energy was −3.1 eV, which is higher than that of the other values obtained for the hollow site, top site and bridge site. The calculations therefore indicate that the Zn metal atoms more preferentially attach at the edge site. To obtain the charge distribution associated with this interaction, Bader charge analysis was performed. The analysis shows that a charge of 0.69 e has been transferred from the Zn metal atom to the nearby carbon atom in the graphene layer. The corresponding electron density difference plot is shown in Fig. 8b.

**Figure 9:**
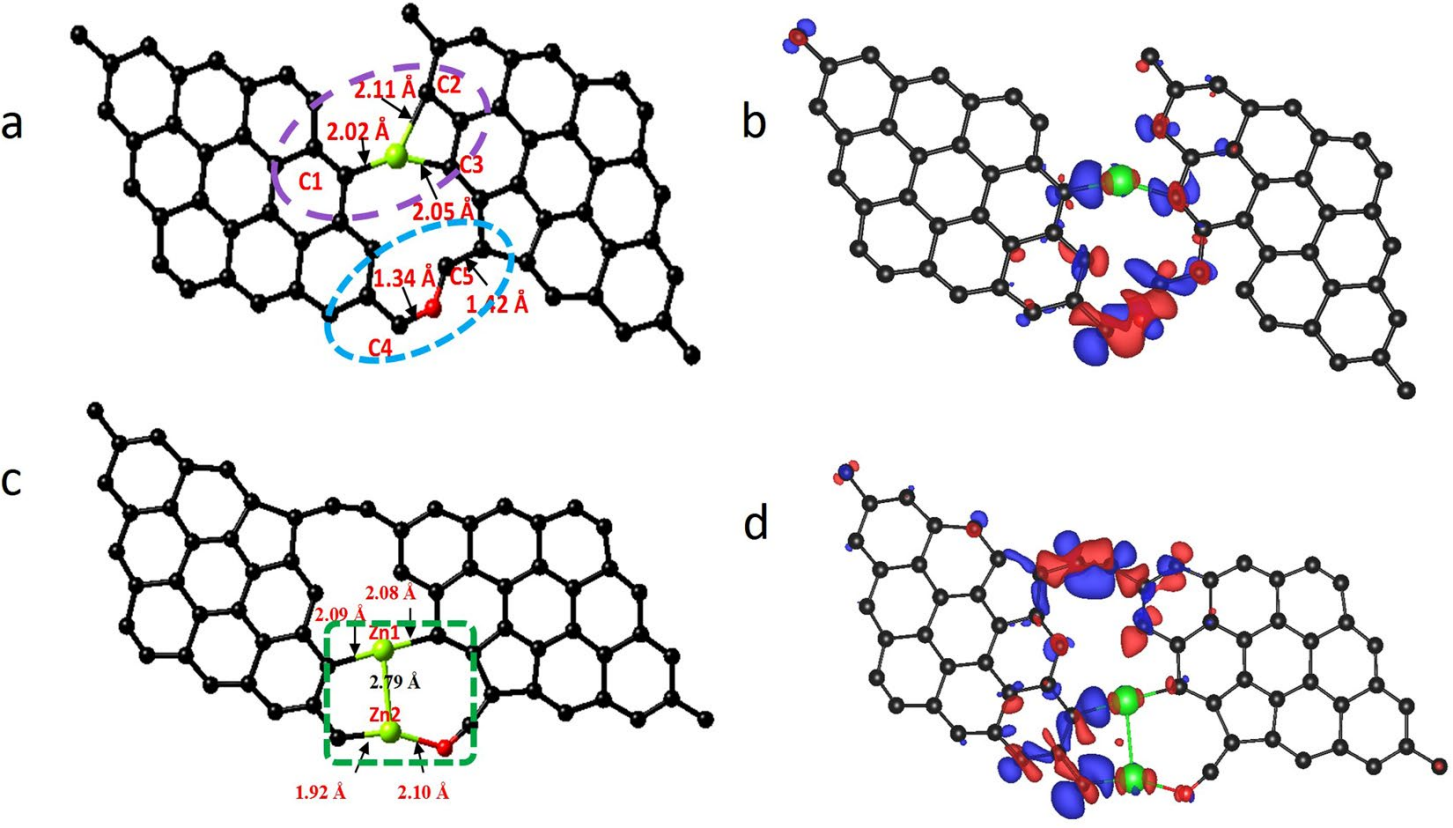
Metal and oxygen atoms incorporated Graphene structure models used for DFT and Bader charge calculations. (**a**) Graphene sheets interconnected through zinc and oxygen atoms with oxygen atom in the next to nearest neighbor position to Zn atom. The electron transfer region is highlighted with violet and blue circles. (**b**) Electron density difference plot of structure given in (a), (c) graphene sheets interconnected with two zinc atoms with oxygen atom in the nearest neighbor position. The electron transfer region is highlighted with green rectangle. **(d)** The electron density difference plot of structure given in (c).

This zinc-edged graphene can interact with the other graphene layer through the zinc atom, and therefore, to understand this interaction, we have constructed a graphene-zinc metal atom-graphene (G-Zn-G) layer configuration, as shown in Fig. 8c, and performed the calculations. The results show an adsorption energy value of −3.2 eV for this configuration. Electronic density difference (Fig. 8d) and Bader calculations show that a charge of 0.62 e is transferred from the zinc atom to the attached carbon atom of the graphene layer, which means that the Zn atom donates a charge of 0.22 e to the carbon (C1) atom of the first graphene layer and 0.45 e to the carbon (C2) atom of the second graphene layer. The calculations indicate that the zinc metal atom is in a position to keep both graphene layers together by transferring part of an electron to each graphene sheet. This means that the Zn atom does not completely transfer the electron to the graphene layer; instead, it tries to establish an electrostatic interaction with both the graphene sheets, and this process helps to grow large graphene sheets in a bottom-up manner. A practical material system has oxygen with graphene, and therefore, calculations were repeated with the inclusion of the oxygen atom into the graphene structure. The initial calculations for oxygen show that among three surface sites (the top site, hollow site and bridge site) the oxygen atom preferably binds with the graphene layer at the bridge site (Supplementary Fig. S17) with an adsorption energy value of −4.98 eV. Calculations of the interaction of an O atom at the edge site have revealed a much higher adsorption energy value of −7.72 eV compared to the bridge site. Upon interaction with the oxygen atom at the edge of graphene, the six-membered carbon ring on the graphene edge is converted into a five-membered carbon ring with the formation of a carbonyl group (Supplementary Fig. S18a). The bond angle of the carbon atoms in the newly formed five-membered ring on the oxygen-edge-decorated graphene is 109.5°, which means that there is a change from sp^2^ to sp^3^ hybridization at the graphene edge. The bond distance between the oxygen atom and carbon atom of the carbonyl group is 1.21 Å. From the charge analysis (Supplementary Fig. S18b), it has been found that the carbon atom of the carbonyl group donates a charge of 1.11 e to the oxygen atom and a small amount of charge to the attached five-membered ring of the graphene layer. With the understanding of the role of oxygen, further calculations have been extended to the zinc- and oxygen-edge-bound graphene-graphene system. Two different positions were considered for the oxygen atom near the Zn atom: the first position is next to the nearest neighbour, and the second is the nearest neighbour. When the oxygen atom is at the next-to-nearest-neighbour site, as shown in Fig. 9a, the calculations show an adsorption value of −3.93 eV for the G-Zn-O2-G system. This value is higher compared to the G-Zn-G system. Similarly, calculations were repeated for the G-Zn-O1-G structure (Fig. 9c) with oxygen in the nearest-neighbour position relative to zinc. In this case, the calculated adsorption energy value is much higher (−7.35 eV) than the previous values. These results tell us that the presence of oxygen, in addition to zinc, has increased the stability of the structure. Bader charge analysis was conducted and showed that a charge of 0.76 e was transferred from Zn to graphene in the G-Zn-O2-G system. Furthermore, it was found that a charge of 1.01 e was transferred from the interacting carbon atoms of the graphene layer to the oxygen atom, as highlighted in Fig. 9a and c. In the case of the nearest-neighbour position of the oxygen atom relative to Zn, 0.5–0.9 e from Zn to the attached carbon atoms of the graphene layer and 0.11 e from Zn to the attached oxygen atom were transferred. The above results tell us that when the oxygen is in the nearest-neighbour position, the structure is more stable. In fact, the EXAFS measurement also confirmed the presence of oxygen in the nearest-neighbour position. In all cases, calculations were performed on stacked layers, and it was found that the structures with oxygen have comparatively higher stacking energy values, which means that the structures that contain oxygen atoms are more favourable for stacking.

The stacked structures and calculated stacking energy values are given in the supplementary information (Supplementary Fig. S19 & S20 and Supplementary Table S4).

### Photocurrent measurements using GQD solid sheets

Graphene-based photon detection has been extensively studied due to its broadband optical absorption covering the UV to far-IR range.

**Figure 10:**
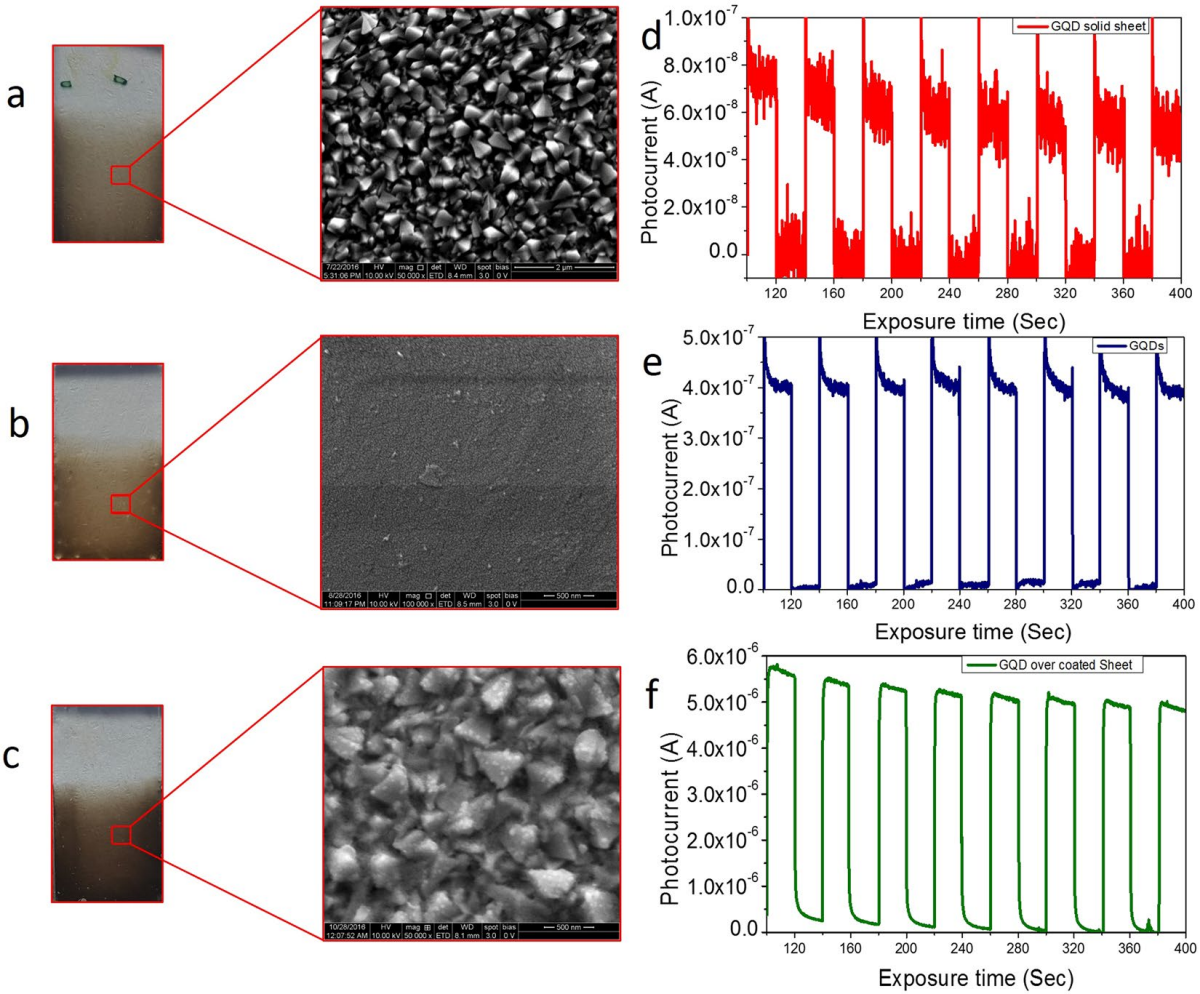
FESEM images and photocurrent curves of EPD coated GQDs and GQD solid sheet structures. The photographic and FESEM images of, (**a**) GQD interconnected solid sheets, (**b**) graphene quantum dots (GQDs), (**c**) GQDs decorated on solid sheets, (**d**–**f**) corresponding photocurrent curves of (**a**,**b** and **c**). The photocurrent measurements show an abrupt raise in the photocurrent upon GQD decoration on solid sheets.

There are many reports on using graphene as an active light-absorbing layer, both individually and in combination with other organic/inorganic systems^45, 58–60^. Here, we present a fully carbon-based photoconducting electrode prepared using GQD solid sheets as active material. The photocurrent measurement experiments were conducted using a three-electrode system, and for this, photoanodes (working electrodes) were produced by the electrophoresis method (electrophoretic deposition details are given in Methods). The Pt wire was used as a counter-electrode and a 0.5 mole of Na_2_SO_4_ as an electrolyte. First, the GQD solid sheets that were separated from the Z7 sample were deposited on the FTO (fluorine-doped tin oxide) substrate (Fig. 10a) for an optimized deposition time of 5 minutes and subjected to photocurrent measurement, which yielded a photocurrent value of 70 nA (Fig. 10d). The photoanodes using the graphene quantum dots (GQDs) (Fig. 10b) were also prepared at different deposition timings (30 to 300 secs in steps of 30 secs), which yielded a maximum photocurrent value of 0.5 µA for 300-sec deposition film (Fig. 10e). To understand the role of the energy transfer mechanism in the photocurrent generation, GQD-decorated solid-sheet electrodes were prepared by the electrophoresis method by depositing GQD film for 30 sec onto the 5-min deposited QD solid film. Interestingly, we noticed a sharp rise in the magnitude of the photocurrent (1.5 µA) from this hybrid structure, with poor coverage of GQDs. When the growth duration of overcoating GQD film was increased in intervals of 30 sec, there were corresponding increases in the photocurrent value. We obtained a maximum photocurrent of 8 µA (Fig. 10f) by growing the GQD layer for 150 secs (Fig. 10c). Since it has been confirmed from the optical study that the photoexcited GQDs are transferring the excitons to the solid-sheet system, we strongly believe that the excitons that are available in the GQD solid-sheet system are converted into useful current in the photoanode. When the coverage was more extensive, a higher number of domains were excited simultaneously, and thus the excited electron delocalization effect was reduced. Once the photoexcited electrons are transferred to the FTO electrode, the holes will be regenerated through the electrolyte by the supply of electrons from the counter-electrode.

## Discussion

GQD solid systems have been successfully prepared by a metal-atom-assisted bottom-up approach using the hydrothermal method. The experimental and quantum chemical calculations have confirmed the solid-sheet performance and formation, respectively. Since we have used zinc chloride as the base substance, a question naturally arises about the role of the Cl atom in the growth process. Cl will preferentially bind to the edges of the graphene, forming a covalent bond with a carbon atom^61^. Cl can also intercalate between graphene sheets and establish interactions with the basal-plane carbon atoms by introducing hole charge carriers into the network. In fact, it is this property of chlorine that is used to exfoliate the graphite into few-layer graphene. There are several reports in the literature on the use of Cl compounds as exfoliating agents^62, 63^. XPS, XRD, FTIR and Raman spectroscopies were used to monitor the interaction of Cl with the graphene system.

Here, in our work, we have noticed the XPS peaks at 201.0 eV and 201.97 eV, which correspond to Cl 2p3/2 and 2p1/2, respectively^64^. They were mostly attributed to the covalent-type interaction with unsaturated carbon at the edges of the graphene sheets. Hence, we ruled out the inclusion of Cl in the graphene network and in the growth process as well. In fact, we have used zinc acetate as a precursor molecule to prepare the solid sheets with same method and confirmed the formation of solid sheets and similar emission behaviour (Supplementary Fig. S15). Therefore, it has been concluded that the electron-donating nature of the metal atoms only helped to grow micron-sized solid sheets.

In summary, we have successfully prepared GQD interconnected solid-sheet nanostructures and characterized their intrinsic optical absorption and emission properties in detail. The solid sheets retained the quantum behaviour of the GQDs, and therefore, it was possible to induce strong emission from them by a pH-independent energy transfer interaction. We were also able to induce strong photocurrent from the GQD solid system through the energy transfer interaction mechanism. By means of X-ray absorption studies, we have confirmed the metal-mediated growth process of the solid sheets. The strong excitonic peak observed from the solid sheets is an evidence for the existence of a quantum confinement effect in our solid sheets. Theoretical calculations have also helped us to confirm the growth process. The emission-producing-type energy transfer interaction and the appearance of strong excitonic peaks from X-ray absorption studies are direct evidence for the formation of band-gap-induced GQD solid sheets. Strong visible-light emission and high photocurrent generation by a resistance-free transport mechanism are all interesting from the point of view of solar cell fabrication. Therefore, we are confident that it will be possible to fabricate the world’s cheapest fully carbon-based solar cells in the near future.

## Methods

### Preparation of graphene quantum dots and GQD solid sheets

All the chemicals were purchased from TCI Chemicals, Japan and used without any further purification processes. Equimolar solutions of citric acid and urea, each 20 ml in volume, were prepared using double distilled water (DDW) and mixed thoroughly by magnetic stirring at 750 rpm for 10 min. After obtaining a clear solution, zinc chloride was added to the solution and the stirring process was continued for an additional 10 min. The concentrations of zinc chloride were 0.0, 0.1, 0.3, 0.5, 0.7 and 0.9 mole for samples Z0, Z1, Z3, Z5, Z7 and Z9, respectively.

After the complete dissolution of all the precursors, the prepared solution looked clear with water-like transparency. This solution was carefully transferred to a Teflon-lined stainless-steel autoclave with a capacity of 40 ml and was maintained at a reaction temperature of 180 °C for three hours in a muffle furnace. After cooling down naturally to room temperature, the samples were collected from the autoclave and stored for further characterization.

### Filtration Process

The filtration procedure was conducted using a dialysis membrane (SPECTRUM LAB products) of 50k MWCO that was purchased from Genetix Biotech Asia Pvt. Ltd. Double distilled water was used as the medium for the filtration processes. The dialysis membrane was activated by soaking it in DDW for 30 min, which was maintained at 90 °C. After activation, the raw sample was filled in the dialysis tube and kept in a beaker containing an excess amount of solvent. The graphene quantum dots, which are smaller than the pore size of the membrane, moved from the solution to the solvent while the solvent was penetrating into the dialysis membrane. The change in colour was observed in the solvent, indicating that the GQDs were filtered out. The solvent was replaced with fresh solvent every 4 hr for a total of 6 times. The filtered-out and filtered-in samples were collected separately and stored for further processing.

### Characterization

HRTEM analysis was carried out using a JEOL JEM-2100 at an operating voltage of 200 kV. The samples were coated on carbon-coated copper grids (200 mesh) and used for the measurements. UV-Vis absorption spectroscopy measurements were carried out using an Agilent CARY 60 spectrophotometer. PL excitation and emission measurements were carried out using a Horiba Jobin Yvon fluoromax-4 spectrofluorimeter. The PL lifeime measurements were carried out by using an IBH time-correlated single photon counting (TCSPC) system, and the decay profile was deconvoluted using IBH data station software V2.6. The samples were drop-casted on glass substrates and used for the XRD and RAMAN measurements. Raman spectra were recorded using a Horiba LABRAM HR excited by a 514-nm laser. X-Ray diffraction measurements were carried out with an Xpert PRO PANalytical instrument. XPS measurements were carried out using the XPS-ion-pumped chamber of an ES-2401 spectrometer equipped with a source of Mg Ka radiation at a photon energy of 1253.6 eV. The Moire pattern was captured by a high-resolution transmission electron microscope (HRTEM; Zeiss Libra 200 FE) operated at 200 kV.

### AFM Characterization

AFM analyses were conducted following the procedure reported by R. Jalili *et al*.^65^ Precisely, the pre-cut silicon substrate was cleaned using an ultrasound cleaner with a mixture of isopropyl alcohol and double distilled water. The silane solution was prepared by mixing trimethoxysilane and double distilled water at a 1:9 volume ratio, and 1 drop of 36 wt% concentrated HCl was added to that solution. The cleaned substrate was then silanized by dipping it for approximately 30 min. Later, the silicon substrate was withdrawn gently from the silane solution and washed thoroughly with DDW. Then, the silanized substrate was dipped into the well-dispersed graphene solution for 5 min. Finally, the graphene-coated silicon substrate was retrieved slowly and vacuum dried at room temperature. The well-dried graphene film on silicon substrate was then subjected to non-contact-mode AFM analysis using an APEResearch instrument.

### Soft X-ray absorption measurements

XAS measurements were carried out in an ultra-high vacuum (UHV) chamber with a base pressure of 5 × 10^−10^ mbar. The measurements were performed in the total electron yield (TEY) mode.

### EXAFS characterization

The EXAFS measurements at the Zn K-edge were carried out at room temperature at the Energy Scanning EXAFS beamline (BL-9) at the Indus-2 Synchrotron source (2.5 GeV, 250 mA) at the Raja Ramanna Centre for Advanced Technology (RRCAT), Indore, India^66, 67^. The EXAFS measurement at the Zn K-edge (9659 eV) was performed in the fluorescence mode by placing the sample at an angle of 45° relative to the direction of the incident beam. For the data collection in the fluorescence mode, an ionization chamber filled with a predetermined gas mixture was used to measure the incident flux (*I*_0_) prior to the sample, while a Si drift detector placed at 90° relative to the incident beam was employed to measure the fluorescence signal (*I*_*f*_) from the sample. The X-ray absorption co-efficient of the sample was determined by µ = [I_f_/I_0_], which was obtained as a function of energy by scanning the monochromator over a specific energy range.

### DFT based Quantum chemical calculations

The computational details of quantum chemical calculations for adsorption energy calculation and charge analysis are given in the Supplementary information.

### Photocurrent measurements

The samples were electrophoretically coated onto a pre-cleaned FTO substrate under a constant potential of 5 V from a DC regulated power supply. Photocurrent measurements were carried out using an LOT-Quantum Design 150 Watt solar simulator with 1 sun illumination attached to a metrohm Autolab PGSTAT 302 N instrument. The EPD-coated samples were used as working electrodes, while the platinum wire and Ag/AgCl were used as counter and reference electrodes, respectively. A total of 0.5 M Na_2_SO_4_ was used as the electrolyte.

### Data availability

The data that support the findings of this study are available from the corresponding author and the same will be provided upon reasonable request.

## Electronic supplementary material

Supplementary Information
