# Supplementary material for: Graphene Quantum Dot Solid Sheets: Strong blue-light-emitting & photocurrent-producing band-gap-opened nanostructures
Source: Sci Rep. 2017 Sep 7;7:10850. doi: 10.1038/s41598-017-10534-4 (PMC5589879; doi:10.1038/s41598-017-10534-4)
Supplement: Supplementary file 1 — Supplementary Information [file 41598_2017_10534_MOESM1_ESM.docx]

**Supplementary information for**

**Graphene Quantum Dot Solid Sheets: Strong blue-light-emitting & photocurrent-producing band-gap-opened nanostructures**

Ganapathi Bharathi^1^, Devaraj Nataraj^1,2#^, Sellan Premkumar^1^, Murugaiyan Sowmiya^3^, Kittusamy Senthilkumar^2,3^, T. Daniel Thangadurai^4^, Oleg Yu Khyzhun^5^, Mukul Gupta^6^, Deodatta Phase^6^, Nirmalendu Patra^7^, Shambhu Nath Jha^7^, Dibyendu Bhattacharyya^7^

1 Low Dimensional Materials Laboratory, Department of Physics, Bharathiar University, Coimbatore, TN, India

2 Centre for Advanced Studies in Physics for the development of Solar Energy Materials and Devices, Department of Physics, Bharathiar University, Coimbatore, TN, India

3 Molecular Quantum Mechanics laboratory, Department of Physics, Bharathiar University, Coimbatore, TN, India

4 Department of Nanoscience and Technology, Sri Ramakrishna Engineering College, Coimbatore, TN, India

5 Department of Structural Chemistry of Solids, Frantsevych Institute for Problems of Materials Science, National Academy of Sciences of Ukraine, UA-03142 Kyiv, Ukraine

6 UGC-DAE Consortium for Scientific Research, Indore, India

7 Atomic & Molecular Physics Division, Bhabha Atomic Research Centre, Mumbai, India

**#e-mail:** [**de.natraj2011@gmail.com**](mailto:de.natraj2011@gmail.com)

**Size distribution analysis of GQDs:**


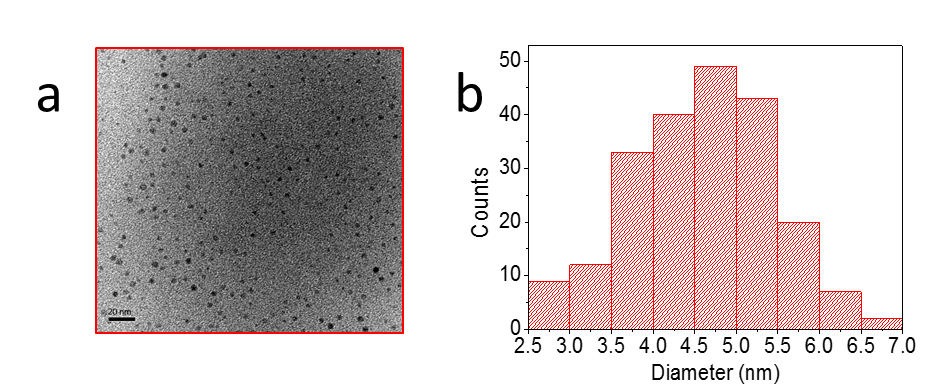


**Figure S1** (a) TEM image and (b) size distribution histogram of graphene quantum dots. The size distribution analysis shows that the average size of the GQDs is around 5 nm.

**XPS analysis:**


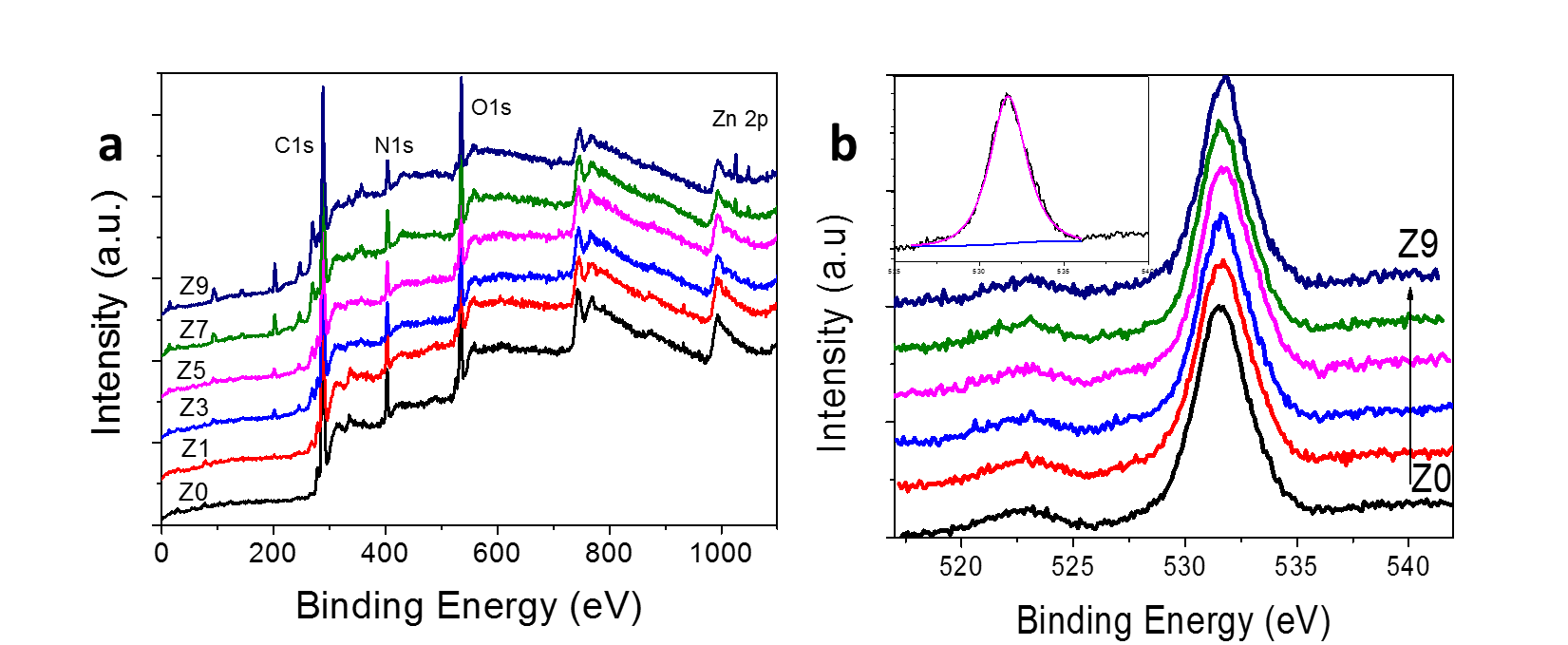


**Figure S2** XPS survey spectra and O1s spectra of GQDs and graphene sheets (a) XPS survey spectra of GQDs and Solid sheet samples (b) symmetric O1s high resolution spectra fitted to a single peak representing the presence of C=O (531.5 eV). Inset shows the deconvoluted spectrum.

Figure 2 XPS spectra of GQDs and graphene sheets (a) deconvoluted high resolution C1s spectra of different sized graphene sheets, gives the evidence of graphitic C=C (at 284.6 eV) with least amount of carboxyl functionalities (at 288.0 eV) (b) symmetric O1s high resolution spectra fitted to a single peak representing the presence of C=O (531.5 eV) (c) N1s high resolution spectra shows broad symmetric peak indicating the major contribution from the graphitic nitrogen components (d) Zn 2p high resolution spectra demonstrates the increase of Zn presence in the graphene sheets analogously incremental to their size.

**XRD analysis:**

**Figure S3** XRD pattern of GQDs (Z0 & Z1) and GQD interconnected solid sheets (Z3-Z9)

Figure S3 shows the XRD pattern of the as prepared samples. The diffraction pattern of Z0, Z1 and Z3 yielded a ‘d’ spacing value of 0.346 nm, which is close to that of an ideal graphite (0.34 nm) and the Z5, Z7 and Z9 samples produced a slightly increased d spacing value of 0.36 nm. This increase in the d spacing value is due to the introduction of oxygen functional groups as discussed in the main text.

**Excitation dependent emission analysis of GQDs:**

**Figure S4** Excitation dependent PL emission spectra of GQDs

**Spectral Overlap between solid sheet excitation (Z7 & Z9) and GQD emission:**


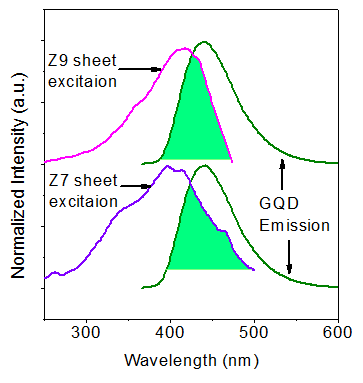


**Figure S5** Spectral Overlap between excitation and emission bands of solid sheet samples (Z7 and Z9) and GQDs respectively. It shows the improved spectral overlapping for Z9-GQD combination.

**HRTEM analysis of Z9 sample:**


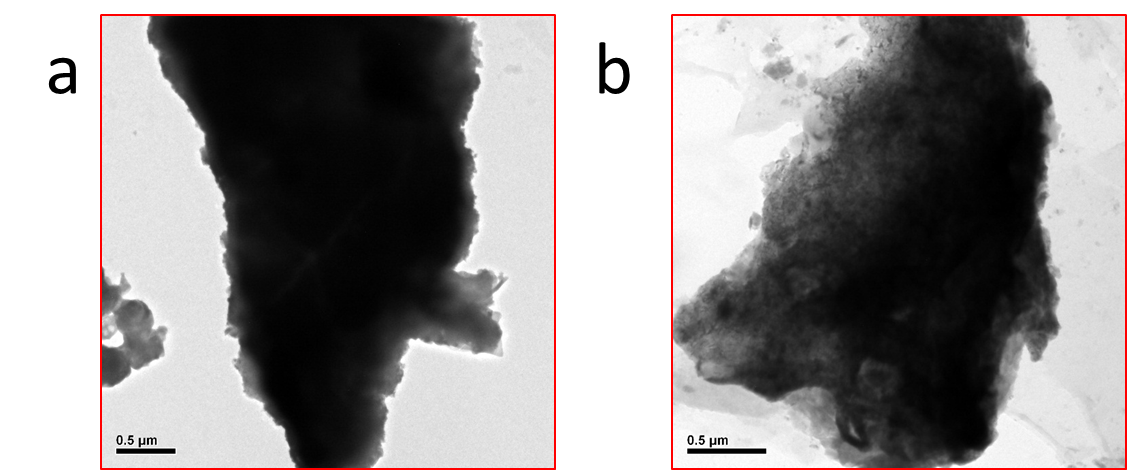


**Figure S6** HRTEM analysis (a, b) TEM images of Z9 sample showing a thick solid sheet of several micrometers in size.

**AFM analysis:**


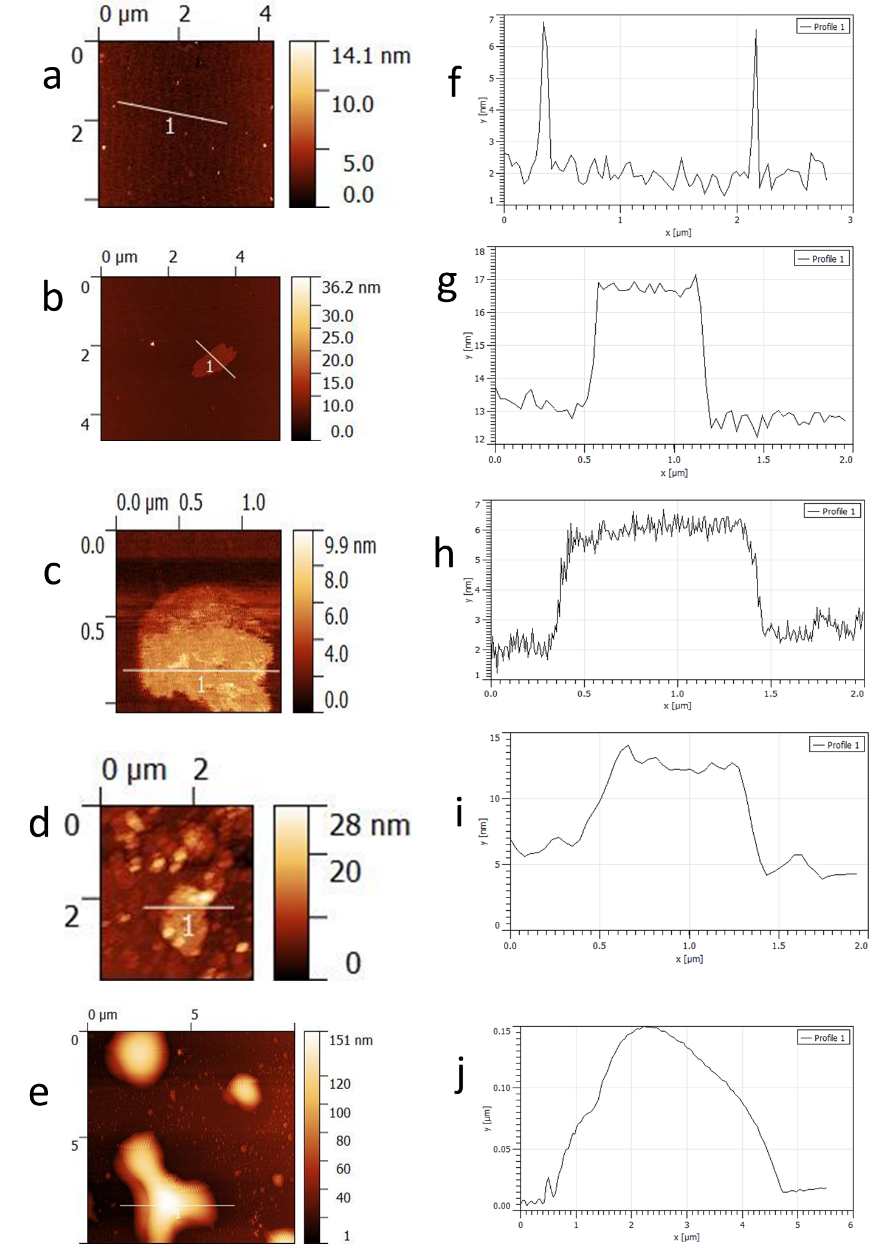


**Figure S7** AFM topographical images and their corresponding line profiles (a-e) AFM images of Z0, Z3, Z5, Z7 and Z9 samples respectively (f-j) line profile analysis of Z0, Z3, Z5, Z7 and Z9 samples respectively

The topographical AFM images are shown in figure S5. A height of about 4 nm was observed for the graphene quantum dot sample, which contains of about 6-8 graphene layers. The average sheet thickness was increased to 8 nm for the Z7 sample (10-12 layers) along with the increase in lateral size to 1 µm. Also, there is an abrupt rise in the sheet thickness for the Z9 sample to 150 nm.


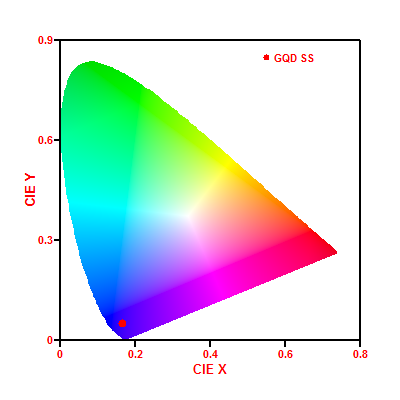
**CIE Chromaticity analysis**

**Figure S8** CIE diagram representing the emission from GQD solid sheets (GQD SS)

**CIE Coordinates**

| Sample | GQD solid sheets |
| --- | --- |
| PL emission maximum | 440 nm |
| x | 0.162 |
| y | 0.051 |
| Purity | 0.91 |
| Actual color | Purplish blue |

**PL decay curves of GQD-solid sheets and ZnS QD-solid sheets:**


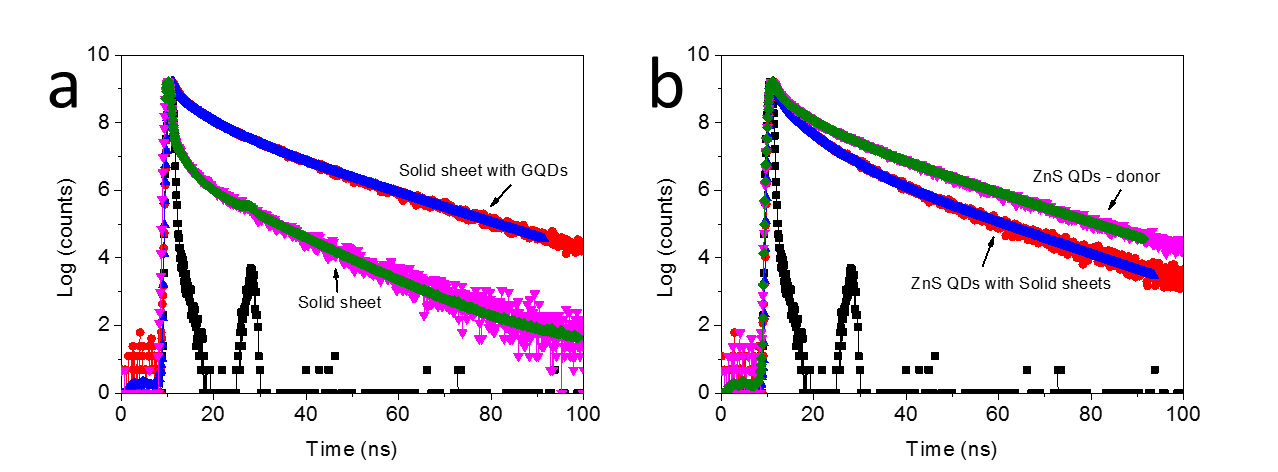


a

**Figure S9** PL decay curves (a) PL decay curves of GQD solid sheet (green line) and solid sheet with GQDs mixture (blue line) measured at 440 nm (b) PL decay curves of ZnS QDs (green line) and solid sheet with ZnS QDs mixture (blue line) measured at 400 nm.

**Photoluminescence properties of GQD interconnected solid sheets and with added ZnS Quantum Dots:**


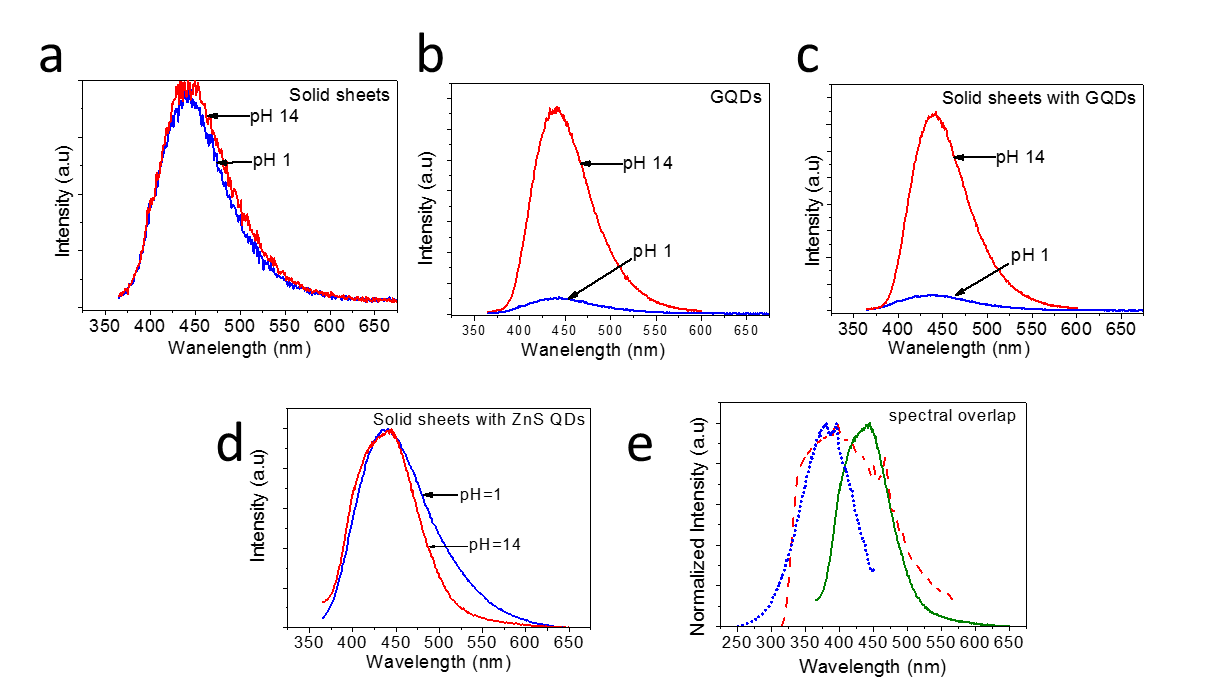


**Figure S10** pH dependent PL analysis of (a) solid sheet sample, (b) GQDs, (c) solid sheets with GQDs (d) solid sheets with ZnS QDs and (e) spectral overlap between ZnS QD emission (red dashed line) and solid sheet excitation (blue dotted line) along with solid sheet emission when mixed with ZnS QDs (green solid line).

The broad emission of ZnS QDs (Fig. S10e) is due to the radiative recombination of the electron-hole pairs trapped in the defect states. This defect related emission is overlapping with the excitation spectra of the GQD interconnected solid sheets which in turn induced strong emission from the solid sheet sample (separated from Z7). The pH independent emission from the ZnS QD-solid sheet mixture is shown in figure S10d.

**Excitation dependent emission analysis of solid sheets:**

**Figure S11** Excitation dependent PL emission spectra of solid sheets

The excitation dependent emission shown in Fig S11 reveals that the emission from solid sheet is however excitation energy dependent, when it was excited directly. This excitation dependent emission behaviour clearly tells us a fact that the solid sheets consist of different sized sp^2^ domains**.**

**Moire pattern observed from Z7 solid sheet sample:**


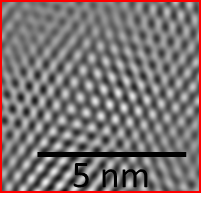


(a)

**Figure S12** HRTEM image showing the presence of moire patterns in our samples (Z7 sample).

Moire patterns are the crystal defects that occur in layered structures due to the orientation mismatch of layers on the bottom up growth process. In graphene, different moire patterns based on the angle of orientation mismatch, were observed and reported. The HRTEM image represented above is similar to the reported pattern[^1^](#_ENREF_1) with crystal orientation mismatch of 4^o^. This observation indicates a fact that there are twists in the as grown solid sheets and this is in addition to the twinning effect in our solid sheet samples.

**Raman analysis of solid sheet samples:**

**Figure S13** Raman spectrum of GQD solid sheet sample separated from Z7 raw sample.

**Elemental mapping analysis:**


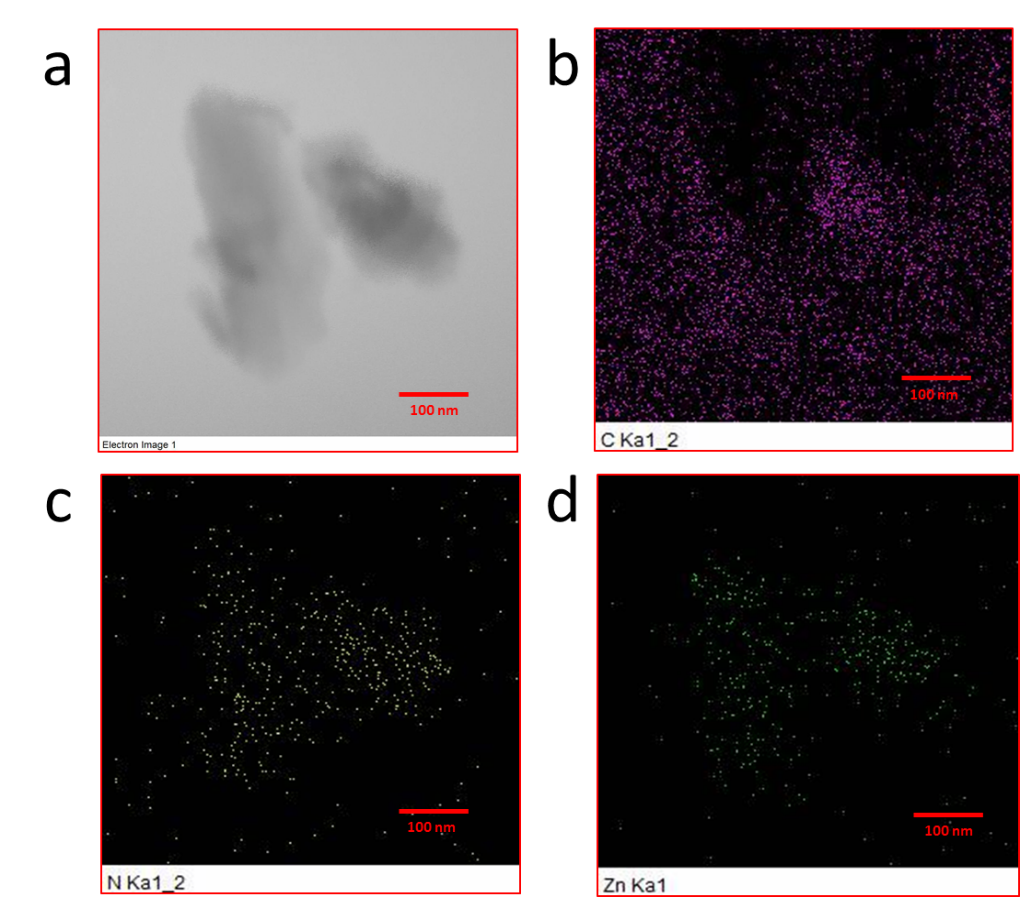


**Figure S14** Elemental mapping analysis (a) electron image of the GQD solid sheet sample (Z5), (b-d) elemental mapping images of solid sheet sample representing the distribution of carbon (violet-d), nitrogen (yellow-e) and zinc (green-f) atoms respectively. It revealed a fact that zinc metal was distributed as individual atoms and no cluster like formation was observed.

**Photoluminescence analysis of samples prepared using zinc acetate:**


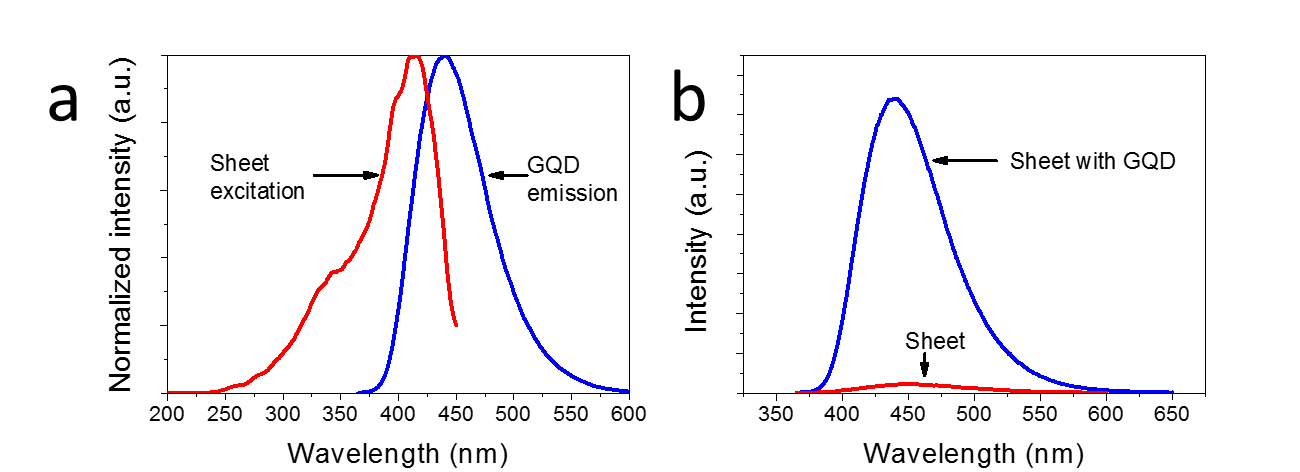


**Figure S15** PL analysis of solid sheets and GQDs prepared using zinc acetate precursor molecule

Fig. S15a shows the spectral overlap between solid sheet (0.7 mole zinc acetate) excitation and GQD emission and Fig. S15b shows the emission comparison between sheet alone and sheet coupled with GQDs samples. It gives out an emission yield of 34% similar to that samples prepared using zinc chloride metal precursor. The above results indicate the similarity of the samples prepared using zinc chloride and zinc acetate precursors.

**Table S1** Excitation, emission and PL quantum yield values of GQDs, GQDs with solid sheets and Solid sheet alone samples

| **Sample** | **Excitation** | **Emission** | **Quantum yield** |
| --- | --- | --- | --- |
| Graphene Quantum Dots (GQDs) | 350 nm | 440 nm | 18 % |
| GQDs with solid sheets | 350 nm | 440 nm | 36 % |
| Solid sheets | 410 nm | 440 nm | 2-4 % |

**Table S2** Values of coordination number, Bond length and Debye-Waller factor obtained from EXAFS analysis at Zn K-edge.

| Sample | Scattering paths | | | | | | | | |
| --- | --- | --- | --- | --- | --- | --- | --- | --- | --- |
|  | Zn – C (1.91Å) | | | Zn – O (2.10Å) | | | Zn – Zn (2.77Å) | | |
|  | C.N | R (Å) | σ^2^(Å^2^) | C.N | R (Å) | σ^2^(Å^2^) | C.N | R (Å) | σ^2^(Å^2^) |
| 6% Zn | 4 | 2.00 ± 0.01 | 0.003 ±0.002 | 1 | 1.90  ±0.01 | 0.002±0.001 | 1 | 2.63 ±0.02 | 0.002 ±0.001 |
| 8% Zn | 4 | 2.03 ± 0.01 | 0.002  ±0.002 | 1 | 1.96 ±0.02 | 0.002 ±0.002 | 1 | 2.65 ±0.01 | 0.015 ±0.008 |
| 10% Zn | 4 | 2.07 ± 0.01 | 0.002  ±0.002 | 1 | 2.30 ±0.01 | 0.027 ± 0.009 | 1 | 2.77 ±0.03 | 0.005 ±0.004 |
| 15% Zn | 4 | 2.01 ± 0.01 | 0.002 ±0.003 | 1 | 2.04 ±0.02 | 0.002 ±0.002 | 1 | 2.60 ±0.02 | 0.008 ±0.004 |

**Computational Details for the Quantum chemical calculations:**

The density functional theory (DFT) calculations were performed using the projector augmented wave (PAW)[^2^](#_ENREF_2)^,^[^3^](#_ENREF_3) method as implemented in the Vienna Ab initio Simulation Package (VASP)[^4-6^](#_ENREF_4). The exchange-correlation interactions were treated by generalized gradient approximation (GGA) with Perdew-Burke-Ernzerhof (PBE)[^7^](#_ENREF_7)^,^[^8^](#_ENREF_8) functional. Van der Waals interactions play a crucial role in the interaction between adsorbate and the graphene layer.[^9^](#_ENREF_9) Therefore, to include the long range interaction between adatom and graphene surface, the PBE functional with Grimme’s^[10](#_ENREF_10)^ D2 correction was used for all calculations. In this method, a semi-empirical dispersion potential was added with the conventional Kohn–Sham DFT energy obtained from PBE functional. In the present study, we utilized a 4x4 hexagonal graphene supercell containing 32 carbon atoms, which was allowed to relax until the total energy difference between the loops was less than 10^-5^ eV and the force was less than 0.02 eV/Å. Previous study has shown that the use of 600 eV cut-off energy and a 6x6x1 k-point grid was sufficient for calculations with 4x4 hexagonal graphene supercell.[^11^](#_ENREF_11) Hence, in all the DFT calculations, we have used a plane-wave expansion cutoff energy of 600 eV and 6x6x1 Monkhorst-Pack[^12^](#_ENREF_12) k-point grid for sampling the Brillouin zone. To accelerate the convergence, a Methfessel-Paxton smearing of the Fermi surface was employed with a smearing width of 0.2 eV. A vacuum region of 12 Å is used to avoid periodic image interactions between the atoms in the supercell.

**Adsorption Energy:**

The interaction between graphene surface and the adsorbate atoms are characterized through adsorption energy,$\text{E}_{\text{ads}}$ and is calculated by using the following equation:

$$\text{E}_{\text{ads}}=\text{E}_{\text{gra}+\text{X}}-\text{E}_{\text{gra}}-\text{E}_{\text{X}}$$

Where, $\text{E}_{\text{gra}+\text{X}}$ is the total energy of the graphene surface with adsorbate X (X = Zn, O or Zn and O), $\text{E}_{\text{gra}}$ is the total energy of the isolated graphene and $\text{E}_{\text{X}}$ is the total energy of the isolated adsorbate X.

**Charge Analysis:**

The charge transfer between the graphene surface and the adsorbate is studied by analysing atomic charge through the Bader charge analysis method[^13^](#_ENREF_13) and electron density difference plot. The electron density difference plot is obtained by subtracting the sum of charge densities of the isolated systems from the charge density of the adsorbed system.

**Table S3** Calculated adsorption energy and charge transfer from Zn to graphene and graphene to O atom for interaction of p-graphene with Zn and O edge-decorated graphene surface.

| Graphene system | Adsorption energy (eV) | Charge transfer (e^-^) | |
| --- | --- | --- | --- |
|  |  | Zn | O |
| G-Zn-G  G-Zn-O-G | -3.2  -3.93 | +0.62  +0.76 | -  -1.01 |

**Table S4** Calculated stacking distance and stacking energy of p-graphene, G-Zn-G and G-Zn-O-G systems

| Graphene system | Stacking distance (Å) | Stacking energy (eV) |
| --- | --- | --- |
| p-graphene  G-Zn-G  G-Zn-O-G | 3.23  3.19  3.17 | -2.95  -3.12  -3.36 |

**Table S5** Calculated adsorption energy and charge transfer from Zn atom to graphene surface and graphene surface to O atom of Zn and O atoms adsorbed on centre or edge of the graphene surface using PBE-D2 method.

| Graphene complex | Adsorption energy (eV) | Charge transfer (e^-^) | |
| --- | --- | --- | --- |
|  |  | Zn | O |
| Zn-Top  Zn-Bridge  Zn-Hollow  O-Bridge  Zn-Edge  O-Edge | -0.13  -0.13  -0.15  -4.98  -3.1  -7.72 | 0  0  0  -  +0.69  - | -  -  -  -0.95  -  -1.11 |


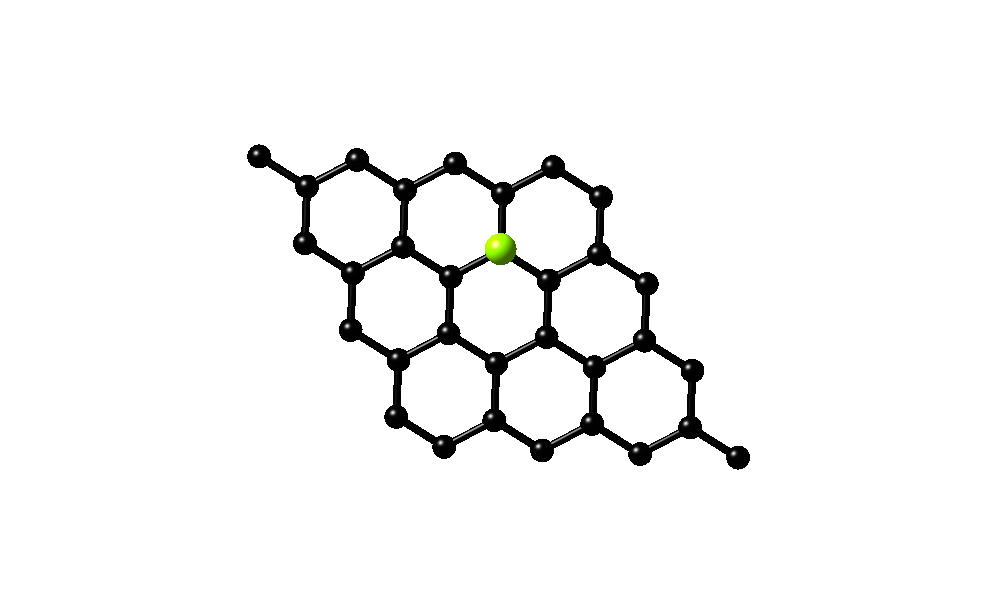

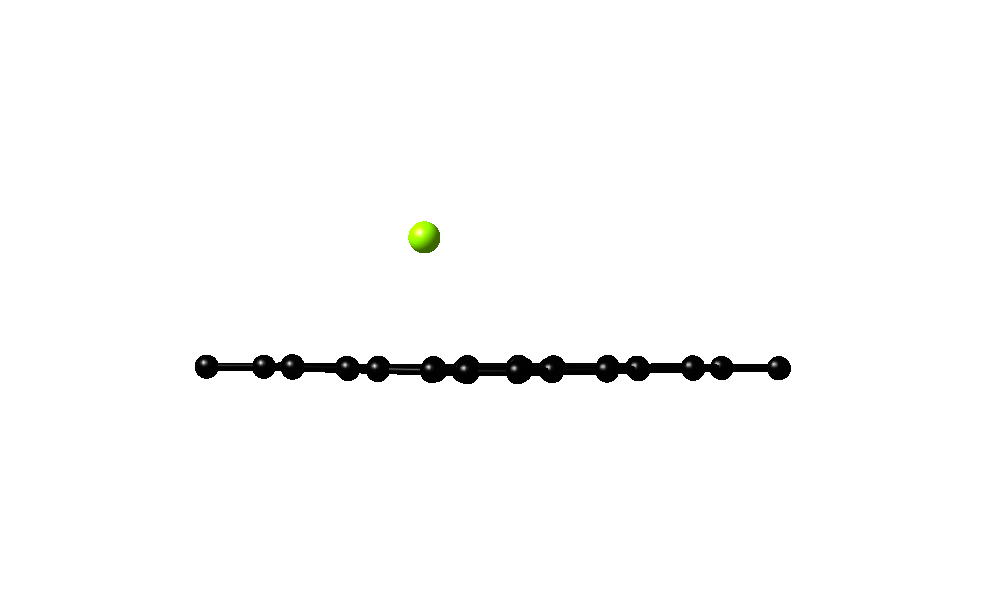


**3.46 Å**

**Top site**


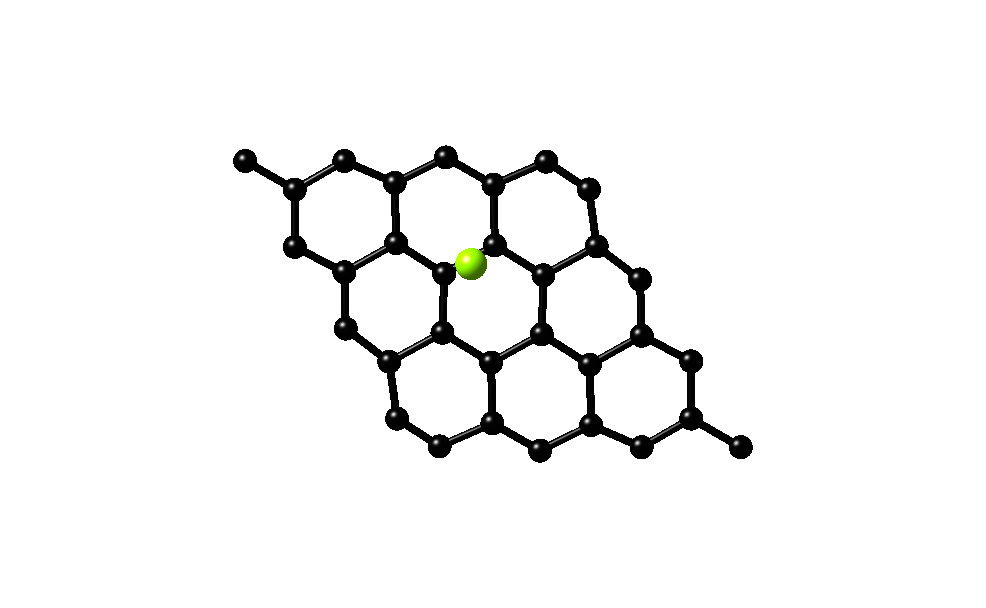

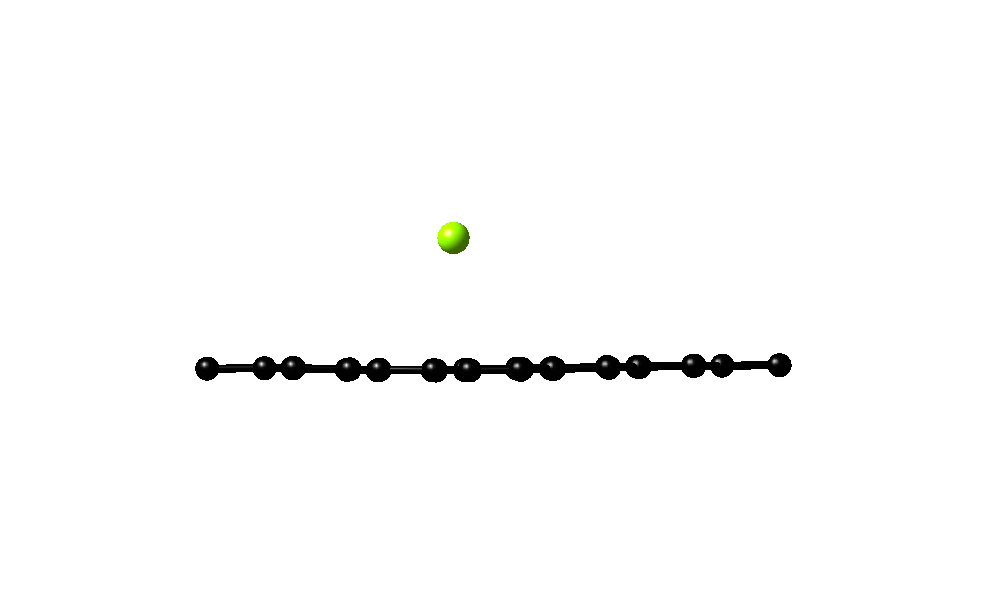


**3.37 Å**

**Bridge site**


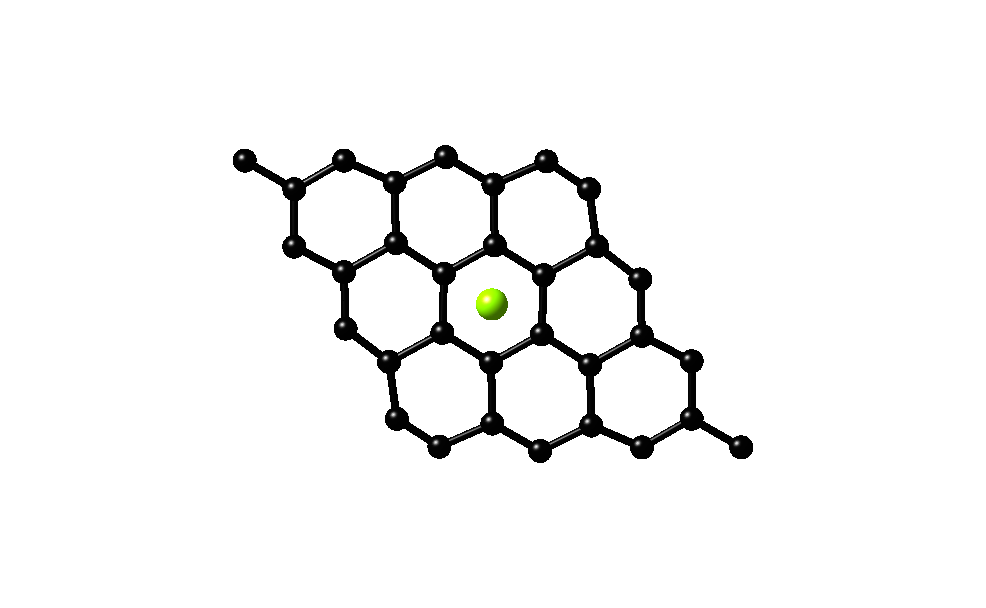

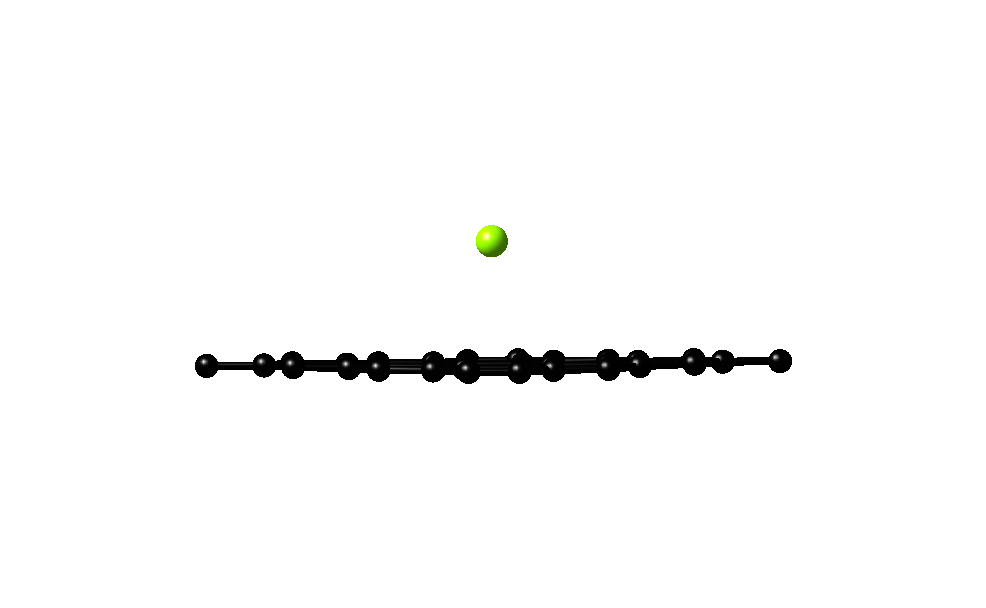


**3.41 Å**

**Hollow site**

**Figure S16**  Top and side views of the optimized geometries of Zn decorated graphene surface. The C and Zn are shown in black and green colours, respectively.


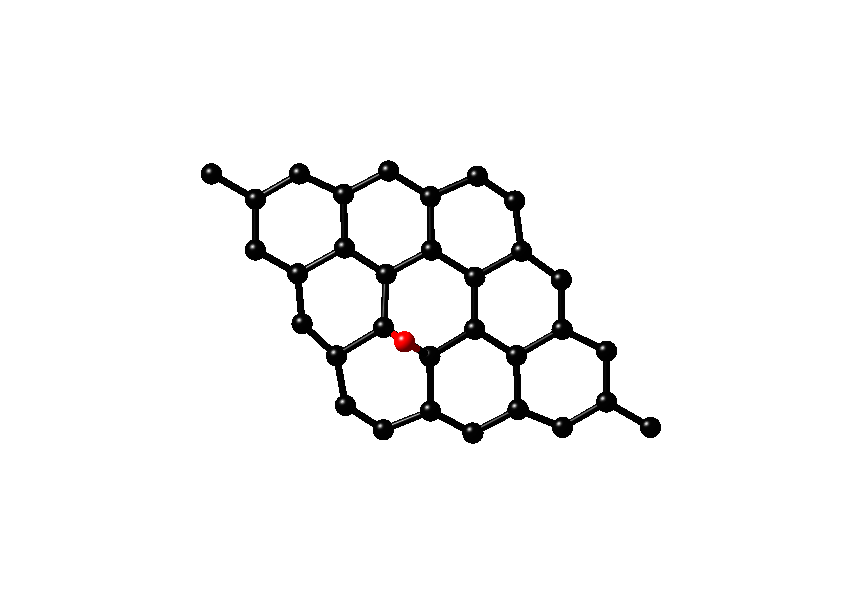

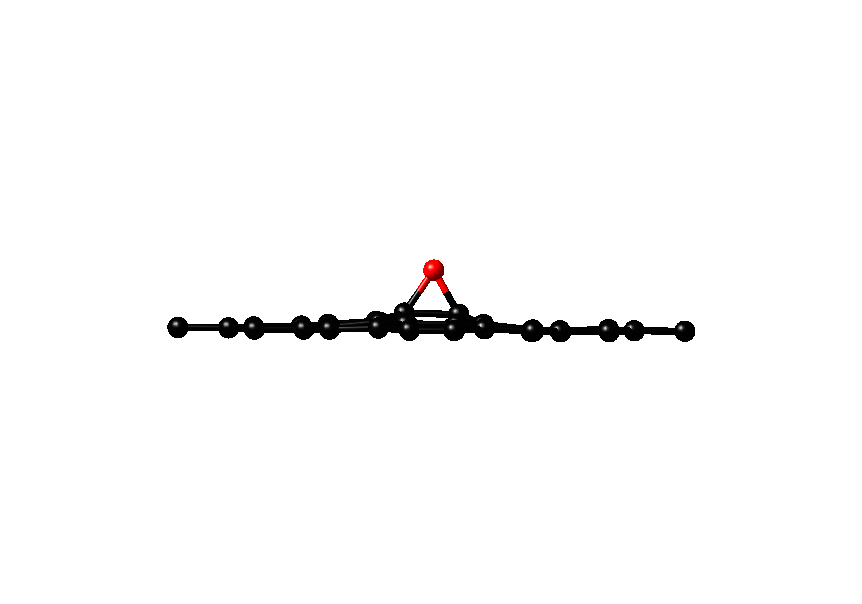


**1.42 Å**

**1.41 Å**


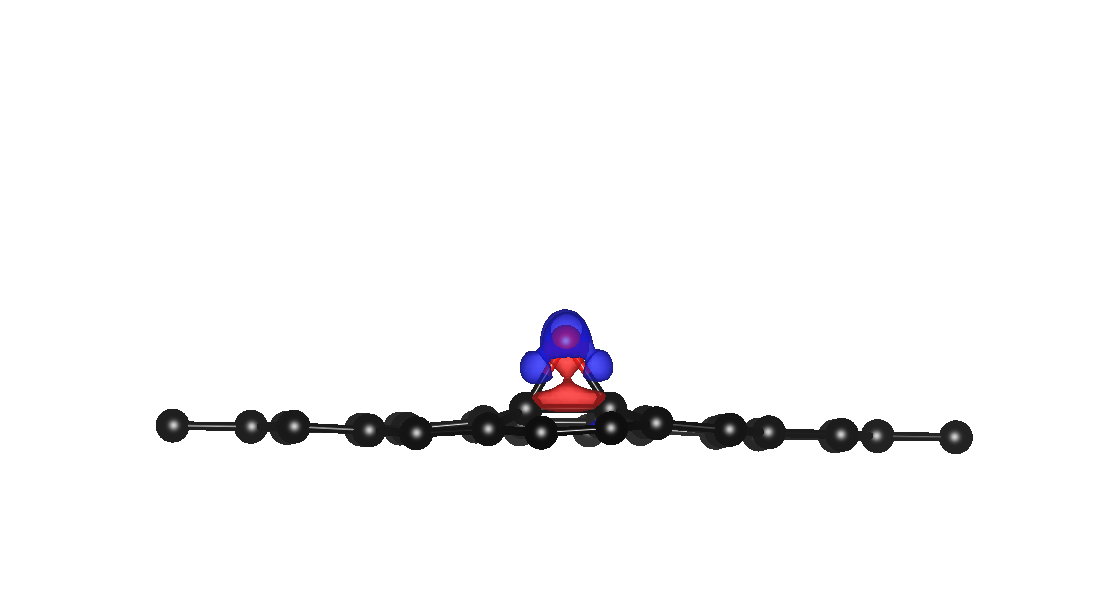


**Figure S17**  Top and side views of the optimized geometry and electron density difference plot of O decorated graphene surface. The O atom is shown in red color. Red and blue iso-surface represent the increase and decrease in electron density, respectively.


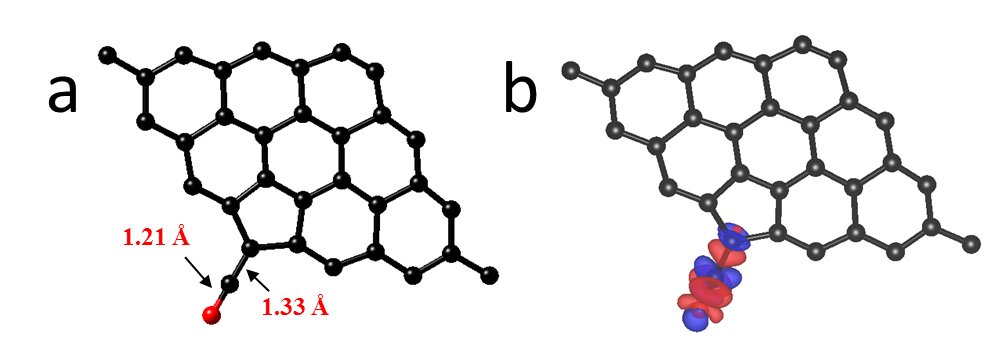


**Figure S18** Optimized geometry of O edge-decorated graphene surface (a) and its Electron density difference plot (b)


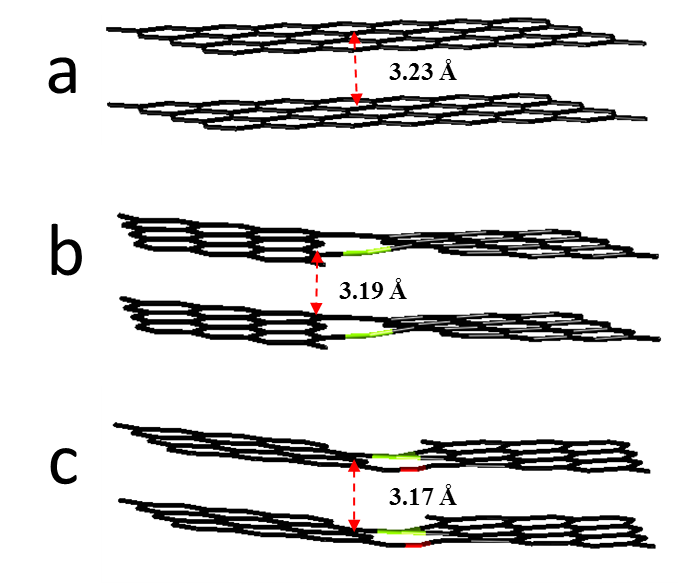


**Figure S19** The stacked structures of (a) pristine graphene, (b) G-Zn-G and (c) G-Zn-O-G


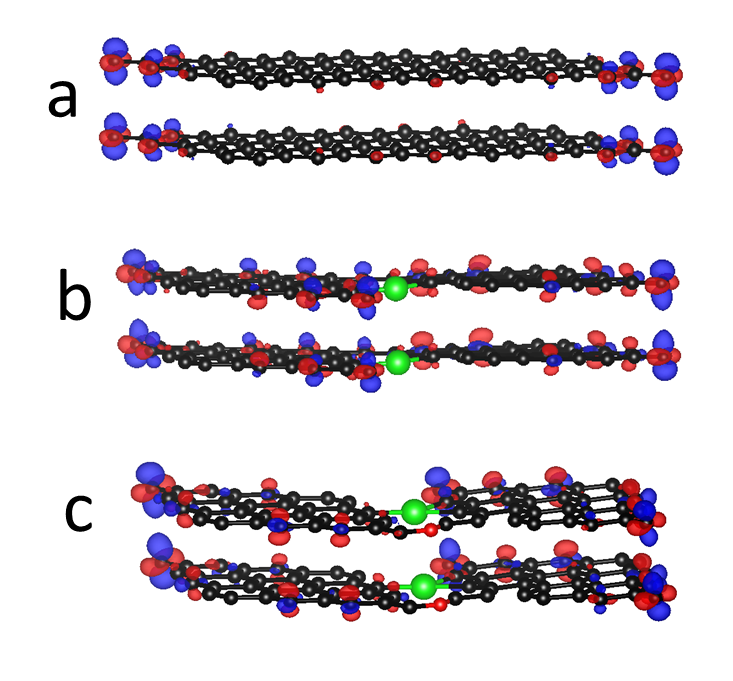


**Figure S20** Electron density difference plot of stacked pristine graphene, G-Zn-G and G-Zn-O-G systems.

References :

1 Miller, D. L. *et al.* Structural analysis of multilayer graphene via atomic moir'e interferometry. *Physical Review B* **81**, 125427 (2010).

2 Blochl, P. E., Jepsen, O. & Andersen, O. K. Improved Tetrahedron Method for Brillouin-Zone Integrations. *Phys. Rev. B* **49**, 16223-16233 (1994).

3 Kresse, G. & Joubert, D. From Ultrasoft Pseudopotentials to the Projector Augmented-Wave Method. *Phys. Rev. B* **59**, 1758-1775 (1999).

4 Kresse, G. & Furthmuller, J. Efficiency of Ab-Intio Total Energy Calculations for Metals and Semiconductors Using a Plane-Wave Basis Set. *Comput. Mater. Sci.* **6**, 15-50 (1996).

5 Kresse, G. & Furthmuller, J. Efficient Iterative Schemes for ab-initio Total Energy Calculations Using a Plane-Wave Basis set. *Phys. Rev. B* **54**, 11169-11186 (1996).

6 Kresse, G. & Hafner, J. Ab Initio Molecular Dynamics for Liquid Metals. *Phys. Rev. B* **47**, 558-561 (1993).

7 Perdew, J. P., Burke, K. & Ernzerhof, M. Generalized Gradient Approximation Made Simple. *Phys. Rev. Lett.* **77**, 3865-3868 (1996).

8 Perdew, J. P., Burke, K. & Ernzerhof, M. Generalized Gradient Approximation MAde Simple. *Phys. Rev. Lett.* **78**, 1396 (1997).

9 Mostaani, E. & Drummond, N. D. Quantum Monte Carlo Calculation of the Binding Energy of Bilayer Graphene. *Phys. Rev. Lett.* **115**, 115501 (2015).

10 Grimme, S. Semiempirical GGA-Type Density Functional Constructed with a Long-Range Dispersion Correction. *J. Comput. Chem.* **27**, 1787-1799 (2006).

11 Liu, X. *et al.* Adsorption and growth morphology of rare-earth metals on graphene studied by ab initio calculaions and scanning tunneling microscopy. *Phys. Rev. B* **82**, 245408 (2010).

12 Monkhorst, H. J. & Pack, J. D. Special Points for Brillouin-Zone Integrations. *Phys. Rev. B* **13**, 5188-5192 (1976).

13 Sanville, E., Kenny, S. D., Smith, R. & Henkelman, G. Improved Grid-Based Algorithm for Bader Charge Allocation. *J. Comput. Chem.* **28**, 899-908 (2007).
